# Cross-Over between Discrete and Continuous Protein Structure Space: Insights into Automatic Classification and Networks of Protein Structures

**DOI:** 10.1371/journal.pcbi.1000331

**Published:** 2009-03-27

**Authors:** Alberto Pascual-García, David Abia, Ángel R. Ortiz, Ugo Bastolla

**Affiliations:** Centro de Biología Molecular ‘Severo Ochoa’ (CSIC-UAM), Cantoblanco, Madrid, Spain; University of California San Diego, United States of America

## Abstract

Structural classifications of proteins assume the existence of the fold, which is an intrinsic equivalence class of protein domains. Here, we test in which conditions such an equivalence class is compatible with objective similarity measures. We base our analysis on the transitive property of the equivalence relationship, requiring that similarity of A with B and B with C implies that A and C are also similar. Divergent gene evolution leads us to expect that the transitive property should approximately hold. However, if protein domains are a combination of recurrent short polypeptide fragments, as proposed by several authors, then similarity of partial fragments may violate the transitive property, favouring the continuous view of the protein structure space. We propose a measure to quantify the violations of the transitive property when a clustering algorithm joins elements into clusters, and we find out that such violations present a well defined and detectable cross-over point, from an approximately transitive regime at high structure similarity to a regime with large transitivity violations and large differences in length at low similarity. We argue that protein structure space is discrete and hierarchic classification is justified up to this cross-over point, whereas at lower similarities the structure space is continuous and it should be represented as a network. We have tested the qualitative behaviour of this measure, varying all the choices involved in the automatic classification procedure, i.e., domain decomposition, alignment algorithm, similarity score, and clustering algorithm, and we have found out that this behaviour is quite robust. The final classification depends on the chosen algorithms. We used the values of the clustering coefficient and the transitivity violations to select the optimal choices among those that we tested. Interestingly, this criterion also favours the agreement between automatic and expert classifications. As a domain set, we have selected a consensus set of 2,890 domains decomposed very similarly in SCOP and CATH. As an alignment algorithm, we used a global version of MAMMOTH developed in our group, which is both rapid and accurate. As a similarity measure, we used the size-normalized contact overlap, and as a clustering algorithm, we used average linkage. The resulting automatic classification at the cross-over point was more consistent than expert ones with respect to the structure similarity measure, with 86% of the clusters corresponding to subsets of either SCOP or CATH superfamilies and fewer than 5% containing domains in distinct folds according to both SCOP and CATH. Almost 15% of SCOP superfamilies and 10% of CATH superfamilies were split, consistent with the notion of fold change in protein evolution. These results were qualitatively robust for all choices that we tested, although we did not try to use alignment algorithms developed by other groups. Folds defined in SCOP and CATH would be completely joined in the regime of large transitivity violations where clustering is more arbitrary. Consistently, the agreement between SCOP and CATH at fold level was lower than their agreement with the automatic classification obtained using as a clustering algorithm, respectively, average linkage (for SCOP) or single linkage (for CATH). The networks representing significant evolutionary and structural relationships between clusters beyond the cross-over point may allow us to perform evolutionary, structural, or functional analyses beyond the limits of classification schemes. These networks and the underlying clusters are available at http://ub.cbm.uam.es/research/ProtNet.php

## Author Summary

Making order of the fast-growing information on proteins is essential for gaining evolutionary and functional knowledge. The most successful approaches to this task are based on classifications of protein structures, such as SCOP and CATH, which assume a discrete view of the protein structure space as a collection of separated equivalence classes (folds). However, several authors proposed that protein domains should be regarded as assemblies of polypeptide fragments, which implies that the protein–structure space is continuous. Here, we assess these views of domain space through the concept of transitivity; i.e., we test whether structure similarity of A with B and B with C implies that A and C are similar, as required for consistent classification. We find that the domain space is approximately transitive and discrete at high similarity and continuous at low similarity, where transitivity is severely violated. Comparing our classification at the cross-over similarity with CATH and SCOP, we find that they join proteins at low similarity where classification is inconsistent. Part of this discrepancy is due to structural divergence of homologous domains, which are forced to be in a single cluster in CATH and SCOP. Structural and evolutionary relationships between consistent clusters are represented as a network in our approach, going beyond current protein classification schemes. We conjecture that our results are related to a change of evolutionary regime, from uniparental divergent evolution for highly related domains to assembly of large fragments for which the classical tree representation is unsuitable.

## Introduction

Structural genomics projects [1] aim at an exhaustive exploration of the space of protein structures realized in evolution [2],[3], speeding up considerably the rate at which new protein structures are resolved. In this context, structural classification of proteins [4]–[9] has become essential for uncovering remote evolutionary relationship that can not be inferred from sequence information alone, and it will have important consequences on our understanding of protein evolution, the sequence to structure to function relationships, the recognition of remote homologs and the modelling of their structures.

This dramatic growth of the number of known protein structures calls upon automatic classification methods that are objective and based only on structural information. The most used structural classifications of proteins, such as SCOP [4] and CATH [5], are manually curated, and therefore they are slow to update. For instance, the last update of SCOP at the moment of writing this paper took from october 2006 to november 2007 (13 months), and the last update of CATH took from may 2006 to january 2007 (9 months). This makes automatic classifications with similar quality to that of CATH and SCOP highly desirable.

However, this goal raises the question of whether, and up to which point, the classification of protein structures is justified. This question is addressed in this paper, where we ask whether an automatic classification based on an objective similarity measure can be uniquely defined.

Several authors studied the agreement between SCOP and CATH classifications [10]–[13], concluding that an overall agreement exists, but it is not satisfactory from a quantitative point of view. This problem is partially due to the fact that SCOP and CATH differ in the way in which they split the proteins into domains [12], which are the units of protein classifications. Nevertheless, they often classify differently even domains that are defined in the same way. Sam and coworkers [13] found out that more than 25% of the domain pairs classified in the same SCOP fold are not significantly similar under two measures of structure similarity.

The other side of the coin is that several structures classified in different folds present a significant structural similarity due to the presence of common substructures, a fact noted for instance by the group of Orengo and later by other groups [14],[15], which in principle makes multiple classifications possible.

The first and most successful automatic classification of protein domains is the database FSSP [8], which is based on the DALI algorithm [9] and on its structure similarity measure. Though this similarity measure is overall consistent with the CATH and SCOP classifications important differences exist [11],[12]. Other approaches aiming at the automatic classification of protein structures have been recently proposed by Rogen and Fain [16], Sam et al. [17], Zemla et al. [18] and by the group of Sippl [19]. However, the FSSP database and its more recent followers do not address the question to which extent structure classification is possible and unique. This question is the subject of the present paper.

### Is Protein Structure Space Discrete or Continuous?

Some of the above difficulties are related with the very essence of protein classification schemes, which assume that it exists an intrinsic level of structure similarity for defining equivalence classes of protein structures. In SCOP, such an equivalence class is called *fold* [20]. Two proteins are defined to belong to the same fold if they share “the same major number and direction of secondary structures with a same connectivity” [4]. In CATH, the corresponding classification level is called *topology*, defined as “the overall shape and connectivity of secondary structures” [5]. These apparently clear definitions are in practice subject to substantial arbitrariety, first because it is not always clear which secondary structure elements belong to the structural core defining the fold and which ones are regarded as optional “embellishments”, and second because one has to allow a certain extent of structural divergence in the protein core.

The difficulties presented above have led several authors to propose that the space of protein structures is continous [13],[21],[22]. This view is supported by the studies that underline the importance of substructures below the level of the globular domain, such as the autonomously folding units of Tsai et al [23], the loops of standard size (approximately 30 residues) of Berezowski and Trifunov [24], or the recurrent fragments of Tendulkar et al. [25] and Szustakowski et al. [26]. Expanding an old idea by Ohno [27], Lupas et al. [28] proposed that the most ancient folds have arisen through an evolutionary process consisting in assembling polypeptide fragments together. These and similar ideas have suggest that the basic unit of protein classification should be substructures below the domain level, defined by Shindyalov and Bourne [22] as “highly repetitive near-contiguous pieces of polypeptide chain that occur frequently” in a set of non-redundant protein structures. If protein domains can be regarded as a combination of such substructures, the resulting structure space should be seen as continuous rather than discrete.

A similar spirit is present in the approaches of Efimov [29] and in particular Taylor, who proposed to enumerate in a kind of periodic table all possible arrangements of secondary structure elements compatible with simple stability rules [30], consistent with the view that evolution of protein structures proceeds by combining simpler modules, resulting in a continuous structure space.

### Homology and Structure Similarity Are Not Always Consistent

Another basic assumption of CATH and SCOP is that evolutionary relationships at the superfamily level imply structure similarity at the fold level. Although this assumption is most of the times correct, it was observed already in Ref. [31] that sequence divergence beyond ≈40% identity sometimes implies large structural variations. Grishin [32],[33] has monitored several examples in which proteins belonging to the same superfamily diverged to the point where they do not share a common fold under the loose definition given above. Interestingly, many of these fold changes take place together with insertions or deletions of large polypeptide fragments, although an interesting example of secondary structure switching has been reported between two homologues regions of distant related proteins [34],[35]. Viksna and Gilbert [36] recently quantified these fold changes in protein evolution, finding that some of them are relatively common. The occurrence of fold change implies that the classification level based on evolution, as the superfamily, and the classification based on structure, as the fold, should not be necessarily consistent, as already recognized by the group of Orengo [14].

## Results

### Objective Fold Definition and Transitive Property

Given the above, one can ask whether protein classifications entirely based on a quantitative measure of structure similarity are possible at all, and if so to which extent.

In formal terms, a protein fold is an equivalence class of protein structures. Mathematically, an equivalence relationship must possess the three property of symmetry, reflexivity and transitivity. Whereas symmetry and reflexivity are automatically fulfilled by any relationship based on a similarity measure, transitivity is not. For transitivity to hold, every time that 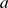 is similar to 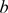 and 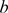 is similar to 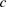, then 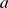 must also be similar to 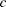. In other words, you can not make a big step from 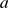 to 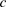 by making an intermediate small step through 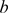. Note that transitivity is not the same as the familiar triangular inequality, 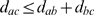, which characterizes similarity measures obtained from a properly defined distance. Rather, transitivity is guaranteed by the much stronger property of ultrametricity [37], 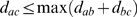, i.e., the distance travelled in two steps can not be larger than the longer of the two steps. An ultrametric set can be uniquely classified in the form of a tree.

#### Uniparental evolution satisfies transitivity

The importance of gene duplication for protein evolution [27] is a reason to expect that protein structural similarity fulfils the transitive property. The distance across the gene tree, i.e., the time spent since the divergence of two genes, is ultrametric (the time spent from the divergence of 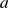 and 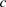 can not be larger than the time either from the divergence of 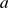 and 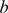 or from the divergence of 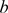 and 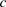), and therefore it is naturally endowed with the transitive property. Therefore, a phylogenetic tree naturally induces a hierarchical classification for every similarity threshold. If pairs of proteins are related through gene duplication, and if their structural dissimilarity correlates with the time of divergence, as it happens for suitable sequence dissimilarities when evolution is neutral, the transitivity property will approximately hold. However, directed evolution where new conformations are positively selected, for instance to fulfill a new function, may violate the last hypothesis.

#### Fragment assembly violates transitivity

Gene duplication is not the only possible mechanism for the evolution of protein domains. Complex proteins are formed from a combination of individual domains with independent evolutionary history. For this reason, the domain and not the complete protein is the basic unit for protein classification. However, there is increasing evidence that globular domains may be formed by combining fragments below the domain level [23]–[26],[28], and it has been observed that many structurally unrelated proteins share common substructures [14],[26],[29]. If two domains 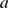 and 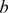 are similar because of a partial substructure 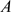, while 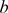 and 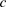 are similar because of a different partial substructure 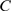, then 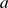 and 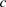 are not similar and transitivity is violated. Several authors refer to this kind of situation by saying that protein space is continuous, since one can connect two different structures 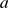 and 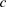 with two small steps passing through 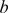.

### Transitivity Violation and Automatic Stop of the Clustering

If 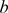 is similar to both 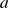 and 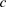 but 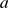 and 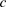 are not similar, there is no classification simultaneously compatible with all the pairwise similarity relationships. Borrowing a term from statistical physics, we can say that the classification problem is *frustrated* [38] when transitivity is violated. We expect that, if this situation is common for many triplets, there is an exponentially large number of substantially different classifications that are almost optimal, in the sense that they violate a small and similar number of pairwise relationships. Conversely, if the transitive property approximately holds, we expect that a well-defined unique globally optimal classification exists, and all sub-optimal classifications are very similar to it.

We expect that the validity of the transitive property strongly depends on structure similarity. Domain pairs with high similarity share most of their structure, and we expect that transitivity approximately holds for them, so that at high similarity the structure space is made of discrete clusters. However, less stringent similarities may be due to partial substructures, and we expect that the transitive property will be violated, and the clustering will strongly depend on the algorithm used.

We propose here a measure to quantify the violation of the transitive property at each step of a hierarchical clustering algorithm. In this way, we aim at detecting the minimum similarity at which transitivity still holds and clustering is justified. At lower similarity, the space should be regarded as continuous, and the significant similarities between clusters should be represented as a network rather than a tree.

Let us consider three elements or clusters 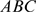, with the convention that 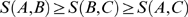. Violation of the transitive property occurs if 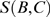 is large while 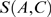 is small, so that 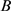 is an intermediate point between 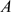 and 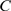. Therefore it is natural to define the transitivity violation of the triangle 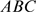 as 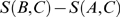. Such a quantity depends on the absolute scale and the offset of the similarity measure, i.e., it is not invariant if we multiply all similarities times a scale factor or we add to them a constant. To remove this dependency, we divide 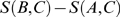 times the difference between the largest and smallest similarities, 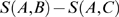, defining the transitivity violation associated to the triangle 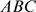 as
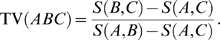
(1)


Notice that, by definition, Eq. (1) is comprised between zero and one because 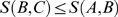 The maximum violation 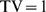 happens when 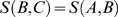 while 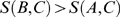.

Another way to interpret this formula is the following. Because of transitivity, only five clustering configurations of the elements 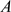, 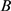 and 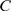 are possible: all elements joined, all separated, two joined and the third one separated. For a threshold 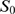, we say that the link 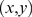 is violated if either 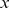 and 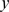 are joined despite 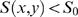 (overunification) or 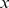 and 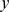 are separated despite 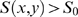 (oversplitting). For thresholds 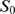 such that 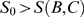 or 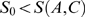 there is one and only one configuration that satisfies all links. However, if 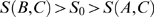, no one of the five possible configurations satisfies all links, since either 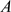 and 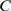 are incorrectly joined, or 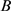 and 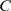 are incorrectly separated. The volume in the space of the threshold parameter 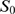 such that some links are violated quantifies the violation of transitivity as 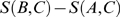. On the other hand, if 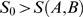 all elements are separated, and if 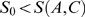 all elements are joined, so that only values of 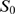 such that 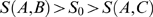 correspond to non-trivial clustering. Therefore, Eq. (1) represents the ratio between the volume of parameter space for which transitivity is violated and the volume for which non-trivial clustering exist.

Yet a third way to look at the above equation is the following. Most hierarchical clustering algorithms join at each step 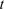 the two most similar clusters 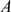 and 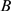 and then recompute the similarity of the new cluster 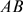 with any other one *C*. For the average linkage algorithm, we use the formula 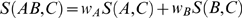, where 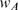 and 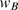 are proportional to the number of elements in sets 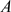 and 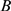. The error made by substituting the original similarities 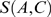 and 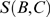 with the combined one is 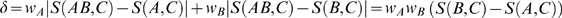, and it is proportional to Eq. (1).

Finally, 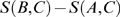 also quantifies the violation of ultrametricity, since in an ultrametric set the two longest sides of any triangle must be equal [37], which implies that 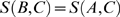. Eq. (1) is normalized in such a way that the value 1 corresponds to the maximum possible violation of ultrametricity, 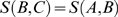.

Now let us consider the step 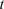 of the clustering algorithm in which clusters 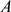 and 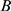 are joined. We define the transitivity violation at this step as the weighted sum of the transitivity violations for all triangles involving 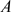 and 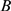:
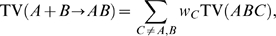
(2)where 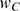 is proportional to the number of elements in cluster 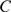, and for each triangle we label as 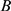 the element such that 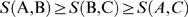.

### Cross-Over in Transitivity Violations

The main result obtained in this study is the existence of a cross-over in the behavior of transitivity violations. This cross-over point determines an intrinsic condition for stopping the hierarchical clustering algorithm. We call the classification obtained at this point “automatic classification”.

The results that we present here are based on a set of 2890 domains that are decomposed very similarly in the SCOP and CATH databases (see Methods), so that the domain decompositions are more likely to be accurate and differences between CATH and SCOP on this set can not be attributed to their different ways of decomposing proteins into domains. We compute structure similarities using the Mammoth-mult algorithm [39], which is one of the fastest algorithms for such a purpose and is comparable in accuracy to other state of the art algorithms [40]. The similarity measure that we use is based on the contact overlap, normalized in such a way as to eliminate the dependence on the domain size for pairs of unrelated domains, and for clustering we use the average linkage algorithm (see Methods). These choices yielded the best results, as described below, and the results presented will refer to them unless otherwise stated.

**Figure 1:**
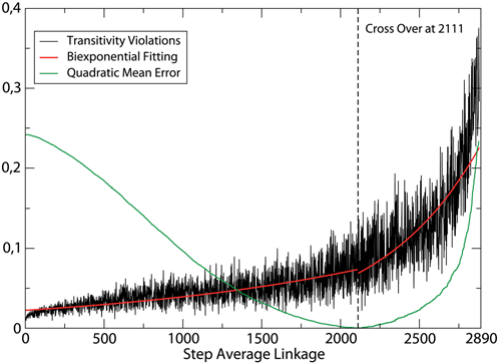
Violations of transitivity, Eq. (2), as a function of the step of the average linkage algorithm. We also plot the mean quadratic error of the two-piece linear fit, whose minimum identifies the cross-over point, plotted as a vertical line;

We plot in Figure 1 the transitivity violations as a function of the step 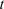 of the clustering algorithm. For large 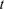 the clusters joined are less similar and the transitivity violations increase. The plot can be divided into two regimes: an initial part with slow increase of transitivity violations at large similarity and a final part with faster increase and small similarity. The cross-over between these two regimes can be detected through a two-pieces fit (see Methods). The normalized error of the fit, plotted in Figure 1 versus the trial cross-over point, allows us to detect at its minimum the optimal cross-over point, depicted as a vertical line. The classification obtained at this cross-over point is called here “automatic classification”, since the threshold similarity at which the clustering algorithm is stopped is automatically determined. We find 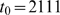, corresponding to joining two clusters with similarity 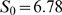. At the stopping point, the automatic classification has 779 clusters.

### Robustness of the Method

In order to test the robustness of our method, we repeated the numerical experiments changing all the relevant choices: The alignment algorithm, the similarity measure and its normalization, the clustering algorithm and the set of domains. In all cases, we observed a clear cross-over in the behavior of the transitivity violations, and the cross-over point could be automatically located through our algorithm. Moreover, the cross-over point did not vary very much for different choices (see Table 1).

**Table 1:** Robustness of the automatic classification.

Set	Ali	Score	Norm	Cl. Al.	N.Clu.	Clus.co.	T.V.	WKSS	WKSF	WKCS	WKCF
SCOP 2890	MM	Cont.	Gauss	AL	**779**	**0.90**	**0.072**	**0.54**	**0.69**	**0.58**	**0.32**
SCOP 2890	MM	**TM**	**No**	AL	740	0.87	0.101	0.59	0.60	0.55	0.22
SCOP 2890	MM	**PSI4-p**	EV	AL	768	0.88	0.088	0.51	0.57	0.51	0.24
SCOP 2890	MM	**PSI6-p**	EV	AL	855	0.87	0.113	0.54	0.58	0.52	0.27
SCOP 2890	MM	**PSI4-t**	EV	AL	788	0.88	0.084	0.49	0.60	0.48	0.26
SCOP 2890	MM	Cont.	**No**	AL	883	0.88	0.069	0.57	0.50	0.53	0.27
SCOP 2890	**MP**	Cont.	**No**	AL	950	0.86	0.070	0.51	0.54	0.53	0.23
SCOP 2890	**MP**	**PSI4-p**	EV	AL	797	0.77	0.089	0.47	0.44	0.49	0.19
SCOP 2890	**MP**	**PSI4-t**	EV	AL	758	0.88	0.085	0.51	0.54	0.51	0.25
SCOP 2890	MM	Cont.	Gauss	**SL**	876	0.90	0.167	0.24	0.48	0.54	0.69
SCOP 2890	MM	Cont.	Gauss	**CL**	730	0.90	0.080	0.26	0.47	0.43	0.10
CATH 2890	MM	Cont.	Gauss	**A**L	776	0.90	0.079	0.50	0.71	0.54	0.36
**SCOP 5041**	MM	Cont.	Gauss	AL	1353	0.92	0.063	0.61	0.52	-	-
**CATH 7073**	MM	Cont.	Gauss	AL	2287	0.91	0.068	-	-	0.51	0.14

The qualitative features of the classification at the cross-over point are robust with respect to different methodological choices. First column, set of domains at less than 40 percent sequence identity: either 2890 domains from SCOP, or the corresponding 2890 domains from CATH, or 5041 domains from SCOP, or 7073 domains from CATH. The number of superfamilies and folds is, respectively: SCOP 2890: 779, 466; CATH 2890: 873, 473; SCOP 5041: 1094, 660; CATH 7073: 995, 1852. 2nd column, alignment algorithm: either the multiple structure alignment algorithm MAMMOTH multiple (MM) or its pairwise version (MP), faster but much less accurate. 3rd column, similarity measures: either Contact Overlap (Cont.) or TM score (TM) or percentage of structure identity (PSI). This can have a tolerance of either 4Å or 6Å , and it can be normalized either with respect the length of the shortest domain, PSI partial (PSI-p), or with respect to the geometric average, PSI total (PSI-t). 4th column, normalization with respect to length: either none, or Gaussian statistics (Gauss) or extreme value statistics (EV) 5th column, clustering algorithms: either average linkage (AL), or single linkage (SL) or complete linkage (CL). The results presented are the following. Number of clusters at the cross-over point (6th column), clustering coefficient averaged until the cross-over similarity (7th column), mean transitivity violations(8th column) and weighted kappa with respect to SCOP superfamilies (9th column), SCOP folds (10th column), CATH superfamilies (11th column) and CATH topologies (12th column), The first line in bold face refers to the selected choices, used in the presented results. In the following lines we evidence in bold face the variables that have changed with respect to the reference.

In order to choose the best options, we measured the transitivity violations, the clustering coefficient, which is the network analogous of the transitive property (see Methods), and the agreement of the automatic classification with SCOP and CATH as assessed through the weighted kappa measure, which is a normalized measure of consistency between two classifications (see Methods). These measures tend to be consistent, i.e., choices yielding larger clustering coefficient tend to yield smaller transitivity violations and larger weighted kappa as well. This justifies the use of the weighted kappa to assess the method, despite the problems that we will discuss in the following and that limit the best possible agreement between the automatic classification and SCOP or CATH. In particular, we considered the following options:

1. As **structure alignment** method, we used either the multiple [39] or the pairwise [41] version of the MAMMOTH algorithm. As it has been recently assessed through an extensive test [40], MAMMOTH multiple is of comparable accuracy to other state of the art structure alignment tools and faster than most of them, while its pairwise version is even faster, but at the expense of accuracy. Moreover, the two algorithms are based on different principles, since Mammoth pairwise optimizes the local superimpositions of heptamers whereas Mammoth-mult optimizes the global superimposition of the two structures. Nevertheless, we obtained very similar results with the two algorithms, which shows that the whole methodology is not very sensitive to the accuracy of the alignment. We used the more accurate MAMMOTH-mult algorithm as the standard option.

2. We used several different measures of **structure similarity**. First, we used measures that require optimal rigid-body superimposition of the aligned residues. Such is the the percentage of structure identity (PSI), which counts the percentage of aligned residues that superimpose within a given threshold after optimal rigid body superimposition. In order to examine the influence of this threshold, we used the standard value 4Å as used in the standard MAMMOTH score and the larger tolerance 6Å. We normalized the PSI either through the length of the shorter protein, Eq. (5), which does not penalize matches that are only partial (we refer to it as the Partial PSI) or through the geometric mean length, Eq. (6) (Total PSI). As an alternative to an arbitrary tolerance parameter we tested the TM score [42], which uses a length dependent threshold that makes this score almost independent of the size of the aligned proteins. Second, we used the contact overlap, Eq. (7), which does not depend neither on the optimal rigid body superimposition nor on a tolerance parameter, although it depends on the parameter used to define contacts, i.e., interatomic interactions in the native structure. Most of the results presented here are obtained with the overlap as similarity score.

In order to remove the dependence on protein length for unrelated proteins, we normalized the PSI and the overlap as in Eq. (8). The parameters used in this expression were determined by fitting mean and standard deviation of the similarity of unrelated structures with respect to the length used to normalize the PSI, using either Gaussian statistics Eq. (9), or extreme value statistics, Eq. (10), as in the original Mammoth paper.

The best similarity score was selected based on the value of transitivity violations and the clustering coefficient evaluated up to the automatic cross-over point (see Methods). Using these criteria, the best score was the contact overlap (see Figure S1).

The normalization with respect to domain size did not modify the clustering coefficient considerably. However, measures that omit the normalization yield much lower agreement with expert classifications, and their cross-over points are rather distinct, whereas all the normalized scores have almost the same cross-over points. Therefore, normalized scores were used as the standard.

3. As **clustering method**, we considered average linkage (AL), single linkage (SL) and complete linkage (CL). We also used the neighbour joining algorithm (NJ), finding results very similar to those with average linkage (data not shown). For this comparison, we did not use the clustering coefficient, since it does not depend on the clustering algorithm.

The plot of transitivity violations for the three algorithms is shown as Figure S2, plot A. Not surprisingly, we found the best results with the average linkage algorithm, which can be interpreted as an algorithm trying to minimize the combination of oversplitting and overunification transitivity violations. The complete linkage only minimizes overunification errors, since it separates all structures that are below the similarity threshold. Its transitivity violations are only slightly larger than for the average linkage, but its weighted kappa is much smaller. The single linkage only minimizes oversplitting errors, since it joins all pairs above the similarity threshold. Correspondingly, it generates larger clusters. Its transitivity error is much larger than for complete and average linkage.

Remarkably, single linkage clustering agrees much better than average linkage with the CATH classification at topology (fold) level. This is not surprising, since CATH uses single linkage clustering, but it is an interesting observation, since it illustrate that one basic difference between CATH and SCOP arises from their reliance on different clustering procedures. However, superfamilies agree much better with the average linkage classification for both CATH and SCOP. More important, the transitivity violation is an intrinsic criterion, not based on any reference classification, which clearly favors the average linkage algorithm (see also the Discussion).

4. As **domain set**, we used the consensus domains (2890 domains), the ASTRAL40 set of domains corresponding to SCOP release 1.63 (5041 domains), and the set of non-redundant domains at the 35 percent sequence identity threshold corresponding to CATH release 3.1.1 (7073 domains).

The number of domains per fold as defined by SCOP (1.67, 2.05) and CATH (1.64, 2.30) increases with the size of the set, as we would expect from the fact that the cluster size is power law distributed, so that smaller samples are more likely to have smaller averages. The same happens at the level of superfamily. In contrast, the number of domains per cluster does not increase for larger samples, being 3.71 and 3.73 for SCOP domains and 3.71 and 3.09 for CATH domains. This indicates that our method tends to stop the clustering process relatively earlier for larger samples. In fact, larger samples are more likely to contain proteins that evidence transitivity violations. The plots of transitivity violations are qualitatively very similar, and are represented in Figure S2, plot B.

### Length Differences

At each clustering step, we measure the difference between the average domain length of the two joined clusters 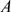 and 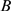,
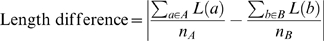
(3)


**Figure 2:**
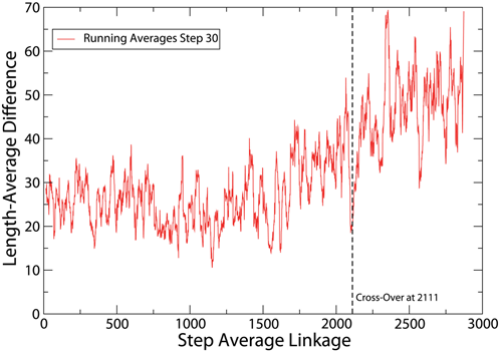
Difference between the mean lengths of the two joined clusters, Eq. (3), versus the average linkage step. The cross-over of transitivity violations is depicted as a vertical line. One can see that length differences are significantly larger after the cross-over. To improve the representation, we performed running averages with window size of 30 steps.

One can see from Figure 2 that the length difference is significantly larger after the cross-over point when transitivity violations increase faster. This observation is consistent with the intepretation that the regime of large transitivity violations takes place when the joined clusters are more likely to share only partial substructures. This behavior of the length difference is very robust with respect to changes in the clustering algorithm, similarity score, or set of domains.

### Statistics of the Cluster Size

At the cross-over point, we find a broad distribution of the number of domains per cluster, with power-law probability density, 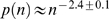. This result agrees with the distribution of the number of proteins predicted to belong to specific folds in various genomes, which follow power-laws [43] with exponents between 2.5 and 4.0, approaching 2.5 for large genomes [44]. It also agrees very well with the automatic clustering by Dokholyan et al. [45], who found an exponent of 2.5 using as similarity measure the Dali score [9], with single linkage clustering and threshold derived from the statistical analysis of the domain similarity network. We also measured the cluster size distribution in the SCOP classification with 40 percent sequence similarity threshold to reduce redundancy, finding 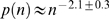 for folds and 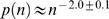 for superfamilies.

Therefore, the exponent of the distribution of the number of domains per cluster agrees reasonably between the SCOP and the automatic classification. Nevertheless, this agreement is not an evidence of the consistency between classifications, since the same size distribution can be found also for clusters obtained from random networks with the same statistical properties [45].

### Comparison of Automatic and Expert Classifications

#### Weighted kappa

**Figure 3:**
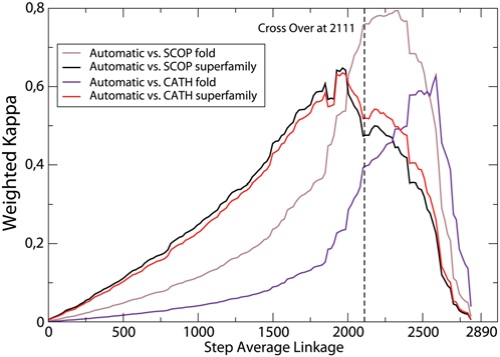
Weighted kappa measuring the agreement the average linkage classifications with step represented in the horizontal axis and SCOP and CATH superfamilies and folds. Notice that the cross-over point, depicted as a vertical line, lies between the maximum agreement with superfamilies and the maximum agreement with folds.

We compared the automatic classification with SCOP and CATH measuring their weighted kappa, which is plotted in Figure 3 versus the step of the average linkage. At first kappa increases steadily, since most joined domains belong to the same superfamily or fold, then it reaches a plateau and it decreases steeply when most of the joined domains belong to different folds or superamilies. The maximum of kappa is reached earlier, i.e., at larger number of clusters, for superfamilies than for folds, as expected since there are more superfamilies than folds. The maximum kappa for folds is larger than for superfamilies, which seem at first sight surprising, since structural similarity is on the average larger within a superfamily than within a fold. However, kappa can be decomposed into the contributions of related and unrelated pairs, with weights proportional to the number of related and unrelated pairs, respectively, see Eq. (20). For folds, the ratio of related to unrelated pairs, and consequently the weight of related pairs, is larger than for superfamilies. Therefore, kappa will be larger when all domains in the same fold are joined than when all domains in the same superfamily are joined.

The cross over point is located before the maximum weighted kappa for folds, indicating that many clustering steps that join clusters containing domains in the same fold imply large transitivity violations. This suggests that these fold relationships are more compatible with a network than with a classification. The difference between the automatic classification and the classification at the step where the kappa for folds is maximum becomes larger when more domains are added to the set, which makes it more likely to find transitivity violations that prevents clusters from being joined.

These results are robust with respect to the different choices mentioned above. In the following, we analyze in more detail the instances of disagreement between the automatic and the expert classifications.

#### Splitting of SCOP and CATH superfamilies

At the cross-over point, the great majority of the clusters only contain domains in the same SCOP or CATH superfamily. Their number is 632 for CATH superfamilies, 664 for SCOP superfamilies, and 673 over 779 (more than 86 percent) for either SCOP or CATH superfamilies (see Table 2).

**Table 2:** Detailed comparison between automatic and expert classifications.

Reference classification	Num. clust.	Homogeneity	Joining probability
SCOP SF	779	85.2	68.0
CATH SF	885	81.1	66.4
SCOP or CATH SF	-	86.3	69.1
SCOP folds	466	92.0	44.5
CATH folds	473	91.4	10.7
SCOP or CATH folds	-	95.4	45.0

First column: reference classification. Second column: Number of clusters in the reference classification. Third column: Percentage of the 779 clusters in the automatic classification that are pure with respect to the reference classification (in case of CATH or SCOP, it is the fraction of clusters that are pure with respect to either CATH or SCOP). Fourth column: Percentage of the pairs joined in the reference classification that are joined in the automatic classification. In the case of folds, only pairs in different superfamilies are counted.

Several superfamilies are splitted in various clusters of the automatic classification. This is one of the most common disagreement between the automatic and the expert classifications. This is however not surprising, since it is well known that evolutionarily related proteins may diverge structurally. The number of splitted superfamilies is 115 over 779 (almost 15%) for SCOP and 87 over 885 (less than 10%) for CATH, which splits several superfamilies that are unique in SCOP.

**Figure 4:**
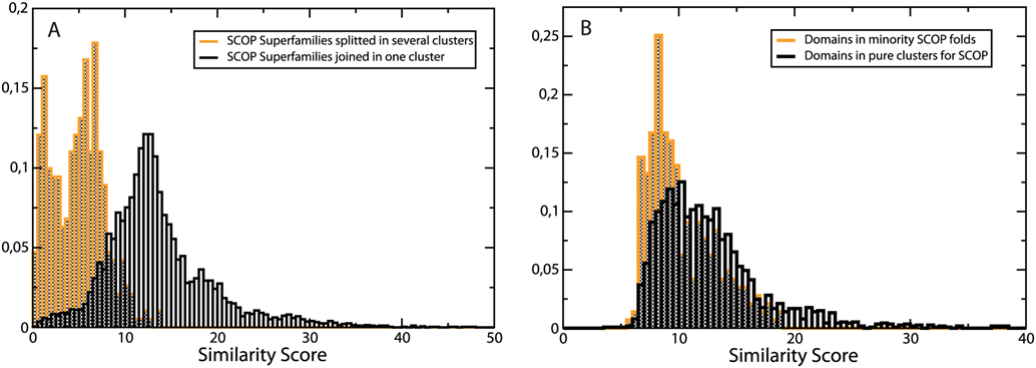
Distributions of intra-superfamily and intra-cluster similarity scores. (A) Distribution of the normalized total similarity score, Eq. (6) and (8), for domain pairs in the same superfamily. The grey bars are obtained for superfamilies that are not split, whereas the white bars are obtained for splitted superfamilies. One can see that splitted superfamilies present a bimodal distribution, with a peak with very small structure similarity. (B) Distribution of the mean intracluster similarity in the automatic classification, Eq. (11). The white bars are obtained for domains in clusters that contain only proteins of the same SCOP fold. The orange bars are obtained for minority domains in clusters containing domains that are mostly of a different SCOP fold.

To analyse these splittings, we measured the distribution of structure similarity between each pair of domains in the same SCOP superfamily, distinguishing split superfamilies from superfamilies contained in just one cluster of the automatic classification. The two distributions are shown in Figure 4A. Similarities in split superfamilies show a bimodal distribution, with one peak at low similarity corresponding to pairs of domains belonging to different clusters and one peak at high similarity corresponding to pairs in the same cluster. This indicates that the splitting is not an artifact of the method, but it reflects a significant difference between split and unsplit superfamilies.

**Figure 5:**
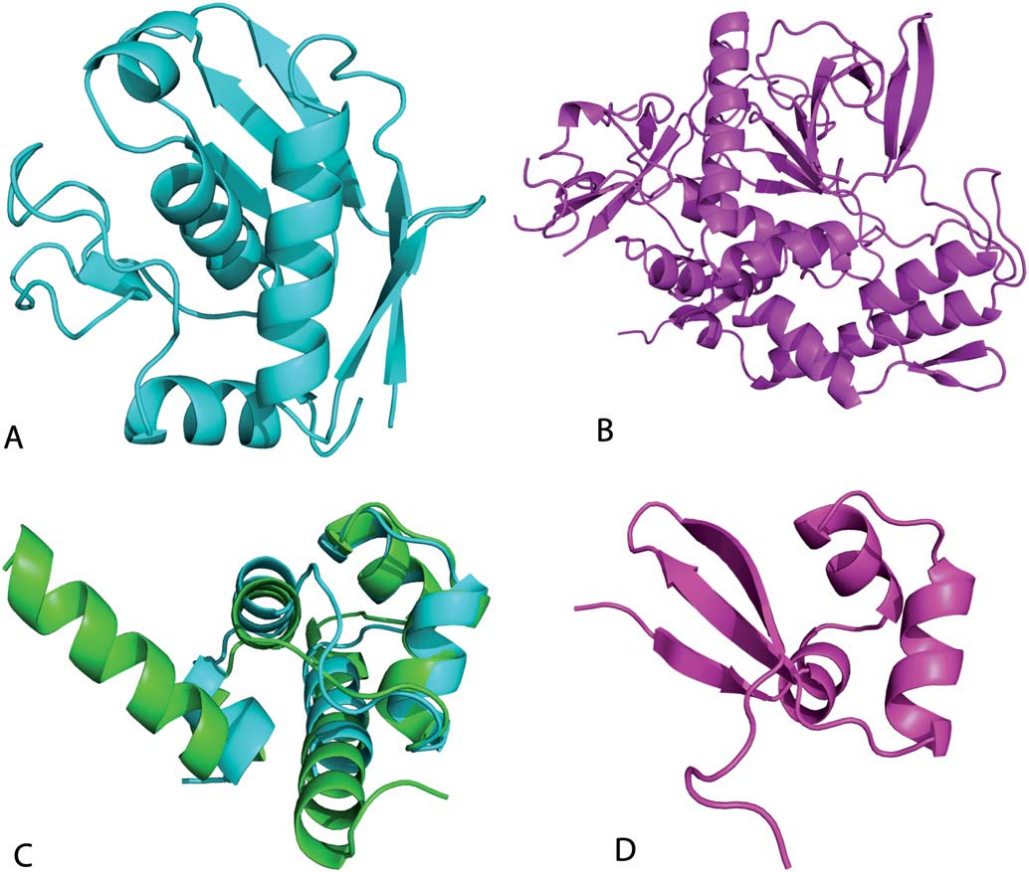
Examples of splitted SCOP superfamilies with large structural changes. Above: Two domains classified in SCOP in the metalloproteases superfamily, but splitted in CATH. Their codes are 1c7ka_ (A) and 1e1h.1 (B), with lengths of 132 and 399 residues respectively. Most of the secondary structure elements in the long protein are not matched in the short one. Below: Lambda repressor-like DNA-binding domains 1lmb3_ and 1r69__ (C) and 1d1la_ (D), which represent a well studied example of possible secondary structure switch in evolution.

For some cases, the difference between domains in the same superfamily appears to be due to large insertions or deletions of secondary structures, which may produce fold changes in protein evolution [32],[33],[36]. In fact, we measured the difference in length between proteins in the same superfamily, distinguishing split and unsplit superfamilies. The median size difference is 41 residues for splitted superfamilies, as compared with 22 residues for unsplitted ones. One such example of split superfamilies is shown in Figure 5A, showing domains 1c7ka_ and 1e1h.1, both from the SCOP superfamily of metalloproteases (55486). The first domain has 132 residues, and it is automatically classified in a cluster of 5 domains from the same superfamily with average length 163. The second domain has 399 residues and it is not joined with any other domain. Only three of the five beta strands in the main sheet of the large domain superimpose with the corresponding strands in the small domain. The large domain has several additional beta strands and alpha helices. CATH also separates the two domains. It includes the cluster containing 1c7ka_ in the superfamily collagenase, and the domain that we separate in the superfamily metalloproteases.

Another example is the superfamily lambda repressor-like DNA-binding domains (47413). We separate this superfamily in two clusters, one containing the domains with ASTRAL id. 1lmb3_ and 1r69__ and another one containing domain 1d1la_. This is consistent with the CATH classification, which separates them in two different topologies, and even two different secondary structure classes (all alpha and alpha+beta). Domains 1lmb3_ and 1d1la_ constitute possibly a very interesting example of evolutionary secondary structure switch between proteins that could be demonstrated to be homologues [34],[35]. Placing both structures in the same fold puts in shadow this very interesting example of divergent structure evolution.

**Figure 6:**
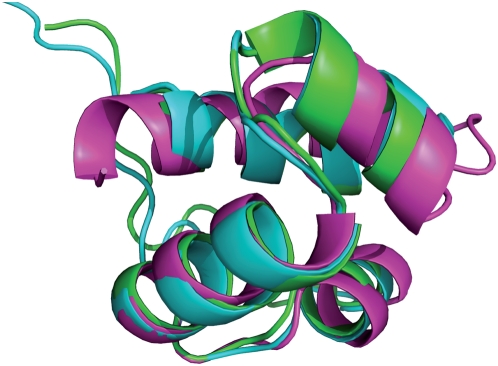
Three small domains of the Homeodomain-like superfamily, with PDB codes 1bl0a1, 1bl0a2 and 1d5ya2 are splitted in two clusters despite very high similarity. These clusters would be joined with 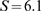, short after the cross-over. This is an example of the limitation of the similarity measure in recognizing significant similarity when dealing with small structures.

A number of splittings is due to the limited ability of the similarity score to assign significant similarity to short proteins In fact, the average overlap or PSI of unrelated structures is larger for short proteins, and therefore a larger overlap or PSI is required to judge it as significant (see Eq. (8)). As a consequence, there is a bias to split superfamilies with small domains: The mean length of splitted superfamilies is 165 residues versus 180 residues for superfamilies that are not splitted. We show one such example in Figure 6, which represents three short domains of the homeodomain-like superfamily that would be joined at a similarity value slightly below the cross-over (at *Z*-score 6.1). A possible solution would be to modify the score so that the similarity does not depend on chain length neither for closely related nor for unrelated proteins. We will study such a modification in following work.

#### Fold unification

The automatic classification disagrees with CATH or SCOP when two domains in the same cluster belong to different folds. This kind of disagreement is rather rare. Only 142 domains over 2890, i.e., less than 5 percent, are contained in clusters where the majority of domains is from another SCOP fold, and they are distributed in only 63 clusters, so that 92 percent of the clusters contains only domains from the same fold. Similarly, 124 CATH domains over 2890 are minority domains, distributed in 67 clusters. However, these do not coincide with the 62 homogeneous clusters according to SCOP. Only 36 clusters (less than 5 percent) are not homogeneous according to both SCOP and CATH, indicating a very high agreement in cluster composition with the expert classifications (see Table 2).

For analyzing these disagreements, we computed the mean similarity score of each domain with the other domains in the same cluster, distinguishing domains in homogeneous clusters from minority domains in clusters with a majority of domains of a different fold. As one can see in Figure 4B, the two distributions overlap quite considerably, but their median values are significantly different, which means that it may be possible to distinguish some minority domains and “clean” some clusters from them. This possible refinement of the clustering will be studied elsewhere.

**Figure 7:**
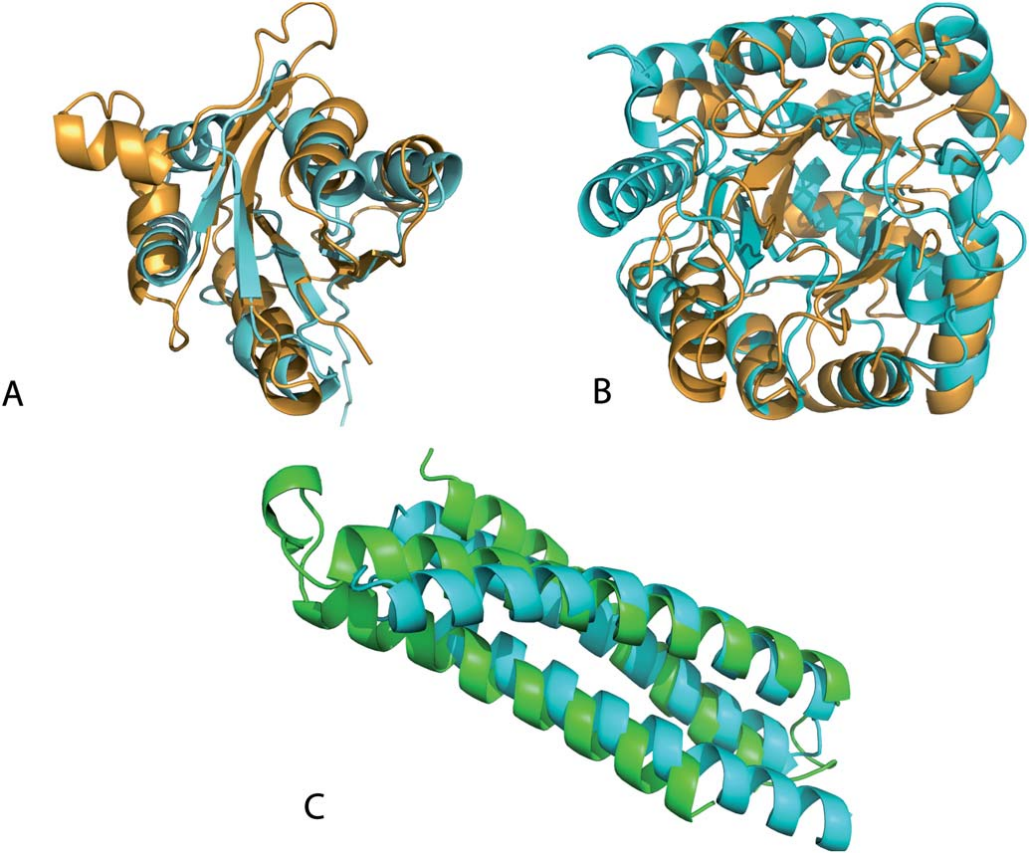
Examples of fold unifications. (A) Domain 1o4wa_ from SCOP fold PIN domain-like and domain 1jmva_ from fold Adenine Nucleotide alpha Hydrolase-like. They have a nearly identical description in the SCOP database in terms of secondary structure elements. (B) The 7-stranded barrel with code 1m65a_ is unified to a cluster with 12 TIM barrel, one representative of which, with code 1j6oa_, is shown for comparison. (C) Unification of two domains from the SCOP folds STAT-like (PDB 1lvfa) and spectrin repeat-like (PDB 2e2aa).

Some examples of fold unification are represented in Figure 7. One such case involves SCOP folds Tim Beta/Alpha Barrel (51350) and 7-stranded beta/alpha barrel (51988). They correspond to two distinct CATH topologies with the same names as in SCOP. However, the distribution of domains in the two folds is not the same in SCOP and CATH. We split these two folds into seven clusters. Four clusters are pure for both SCOP and CATH, which agree in classifying them as TIM barrels, two clusters only contain 7-stranded barrels according to SCOP but all domains but one are classified as TIM barrels in CATH, and the last cluster contains, together with 12 TIM barrel domains, one domain, 1m65a_ that is considered 7-stranded in SCOP and TIM barrel in CATH. Visual inspection supports the 7-stranded classification, in agreement with SCOP, but the structure similarity inside the cluster is very high.

In another example, the automatic classification joins domains from the SCOP folds Spectrin repeat-like (46965, corresponding to CATH topology 12058) and STAT-like (47654, corresponding to CATH topology 1201050) in three different clusters. However CATH classifies domain 1lvfa_, which is STAT-like according to SCOP, in the Spectrin repeat-like fold, while a paper of the SCOP team reports that the SCOP release 1.53 changed the classification of domain 1br0 from spectrin repeat to STAT-like, showing that even experts can confound these two folds [46]. Visual inspection shows that the domains that we unify are indeed very similar.

The third example corresponds to two domains from SCOP folds PIN domain-like (PDB code 1o4wa_) and Adenine Nucleotide alpha Hydrolase-like (PDB 1jmva_), which are automatically classified in the same cluster. Besides a very high structure similarity, these folds have an almost identical description in the SCOP database (beta-sheet of 5 strands, order 32145).

#### Splitting of folds

Another possible disagreement happens when superfamilies that are joined together in the same SCOP fold or CATH topology are splitted in different clusters. This is very frequent: 55.5 percent of the domain pairs in the same SCOP fold but distinct superfamilies are separated. For CATH, this percentage raises to 89.2%. This is not likely to be an artifact of the automatic classification, since the automatic classification agrees with SCOP or CATH at the fold level better than they agree with each other, as discussed in next section. The transitivity analysis suggests that this happens because SCOP and CATH join superfamilies into folds at a similarity level for which transitivity violations are rather large, so that clustering is not justified and unique. At this similarity level different clustering algorithms yield radically different classifications. In contrast, the pairs of domains of the same superfamily that are separated in the automatic classification is significantly smaller, 32% for SCOP and 34% for CATH.

### Analysis of Expert Classifications

#### Comparison between SCOP and CATH

The expert classification schemes CATH and SCOP split proteins into domains differently. Domains in the CATH classification are typically smaller than those in the SCOP classification, with an average of 155 residues compared to 179 residues for SCOP domains (the standard deviations are 88 and 120 respectively). Comparison with a set of expert curated domain decompositions [47] shows that SCOP undercuts domains, whereas CATH decompositions are usually in good agreement with experts [48]. We used here 2890 domains similarly defined in both SCOP and CATH. For this consensus set, we measured the agreement between the SCOP and the CATH classification through the weighted kappa (see Methods). The values found are reported in Table 3, where the automatic classification is also shown for comparison.

**Table 3:** Comparison of the agreement between different classifications.

	Superfam.	Folds
SCOP vs. CATH	0.84	0.48
Automatic (AL) vs. SCOP	0.54	0.69
Automatic (AL) vs. CATH	0.58	0.32
AL (max) vs. SCOP	0.65	0.79
AL (max) vs. CATH	0.64	0.63
Automatic (SL) vs. SCOP	0.24	0.48
Automatic (SL) vs. CATH	0.28	0.70
SL (max) vs. SCOP	0.51	0.67
SL (max) vs. CATH	0.51	0.80

The agreement is evaluated through the weighted kappa parameter, Eq. (19). The first line compares superfamilies and folds from SCOP and CATH. In the two following lines, the automatic classification at the stop point obtained with average linkage (AL) is compared with SCOP and CATH, respectively, at the levels of superfamilies and folds. The two following lines compare the expert classifications with the AL classification at the points where their weighted kappa is maximum. The four last line are the same, but using as clustering algorithm single linkage (SL), which gives a much stronger agreement with CATH than with SCOP at the fold level, consistent with the fact that CATH uses single linkage.

There is rather good agreement, 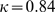, between CATH and SCOP at superfamily level. The 779 SCOP superfamilies become 885 with CATH (almost 14 percent more), but CATH superfamilies are larger, so that 26320 pairs of domains are in the same CATH superfamily versus 22937 for SCOP, of which 90 percent (i.e., 20695) are common.

The agreement with the average linkage clustering is significantly weaker. Around 68 percent and 66 percent of pairs in the same SCOP and CATH superfamily are in the same automatic cluster, since many superfamilies are split in the automatic classification.

In contrast, the agreement between CATH and SCOP at fold level is much poorer, with 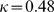. This suggests that the fold is more subjectively defined than the superfamily. The disagreement comes mainly from the fact that CATH joins many more pairs than SCOP at fold level: there are 3.9 times as many pairs classified as same fold and different superfamily by CATH than by SCOP (137608 versus 35428). More than 94 percent of the domain pairs defined by SCOP in the same fold are joined by CATH, but these commonly joined pairs represent only one third of the pairs in the same CATH topology.

Interestingly, at the fold level the similarity based clustering agrees with the two manual classifications better than they agree with each other, with maximum agreement 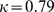 and 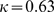 for SCOP and CATH, respectively. At the cross-over point, the agreement between the automatic classification and SCOP is 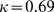, much larger than with CATH 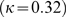.

If we perform the clustering using single linkage instead of average linkage, the agreement between the automatic clustering and CATH becomes much better (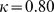 at the maximum and 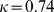 at the stop point), whereas the agreement with SCOP becomes much poorer. Indeed, CATH uses single linkage clustering, i.e., a new domain is joined to the cluster containing the most similar domain if similarity is above a threshold. This explains why CATH joins more pairs of domains than SCOP at the topology level.

If we compare the average linkage with the single linkage clustering as a function of the clustering step, we find that the single linkage joins many more pairs than the average linkage for the same number of clusters, as expected from the fact that it does not penalize the overunification. The weighted kappa between the two algorithms decreases as the clustering proceeds, as shown in Supporting Figure S3. The disagreement between the two classifications is already important before the cross-over point.

These findings shed light on the comparison between CATH and SCOP. Despite their good agreement at the level of superfamily, CATH and SCOP use different criteria for clustering superfamilies. They would nevertheless agree better if the clustering would be stopped at large similarity, where transitivity is approximately fulfilled. Therefore, the discrepancy between CATH and SCOP at fold level has two roots (besides the different in domain decompositions): (1) They use different clustering methods, a procedure effectively similar to average linkage for SCOP and single linkage for CATH. which yields a much larger number of pairs classified as the same fold, despite the number of folds is practically the same. (2) They push the clustering up to a low similarity level at which the two clustering methods diverge considerably.

#### Classification criteria may vary with time

Another possible source of subjectivity in the definition of the fold is the amount of biological knowledge that the expert curators use. To test the influence of this factor, we analyzed how SCOP folds and superfamilies changed through time. We labelled the age of a SCOP fold or superfamily through its SCOP index. Since the SCOP index depends on the secondary structure class, we normalized separately the index for different secondary structure classes, so that a value of 1 means that the index lies within the first 10% of its class and so on. We measured the mean similarity score for pairs of proteins in the same fold or superfamily. The MAMMOTH similarity score of related domains depends on their length. For superfamilies, we find that the average score depends on the average length of the superfamily, 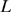, as 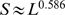. Since the folds and superfamilies with index in the 7th and 8th interval are characterized by much longer domains (the average length is 270, compared with average lengths between 131 and 188 for all other intervals), we normalized the MAMMOTH similarity score dividing it by 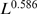, where 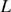 is the average length in the cluster.

**Figure 8:**
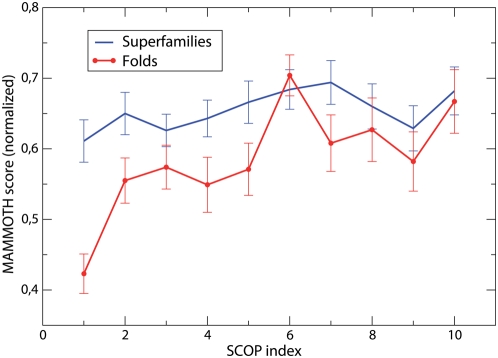
Normalized structural similarity score of the program MAMMOTH (A) and standard deviation of domain length (B) versus the date of the oldest PDB file included in the SCOP fold. Older folds appear to be significantly more structurally diverse, as assessed both through the MAMMOTH score and their length difference.

One can see from Figure 8 that folds classified since longer time (smaller index) tend to be structurally more diverse. They also contain more domains and more superfamilies (data not shown). There are two possible interpretations of these findings. It is possible that some folds are intrinsically more diverse, and that they are more likely to be discovered and studied first, since they contain a larger number of proteins. But it is also possible that the greater biological knowledge available for older folds makes it easier to classify domains in these folds even in the absence of a large structure similarity.

To distinguish between these two interpretations, we measured structure similarity within superfamilies, see Figure 8. Similar as for folds, older superfamilies contain more domains than the more recent ones (11.6±2.2 for the most ancient and 4.1±0.9 for the most recent index interval), but they are not more structurally diverse. This suggests that: (1) Ancient folds are structurally more diverse because they join superfamilies that are more diverse between each other but not within each other. Consistently, ancient folds contain more superfamilies: 3.7±0.8 for folds with the most ancient labels, less than 1.9±0.3 for SCOP labels above the third interval; (2) When there is sequence information to guide the classification, as in the case of superfamilies, the structural diversity remains stable with time, and it does not depend on the size of the superfamily, whereas it changes with time in the case of folds, for which no sequence information is used. This may suggest the existence of a bias to join new superfamilies to a fold known since long time even if the structure similarity is small.

Summarizing, the structure similarity within SCOP superfamilies remained stable through time, whereas the similarity of superfamilies classified into the same fold tends to be lower for ancient folds.

### Beyond the Classification: Protein Similarity Network

The cross-over point of transitivity violations determines an intrinsic threshold beyond which protein similarity is better represented as a network rather than as a tree. Protein similarities have been previously represented as a network by other authors. Dokholyan et al. [45] generated the protein domain universe graph using as similarity measure the Z score of the structure alignment program Dali [9]. They found out that, for proper thresholds, the network is scale-free, i.e., the number of links per node is power-law distributed. Performing single linkage clustering over this network, they obtained clusters whose size distribution is also a power-law, reminiscent of the distribution of protein domains per SCOP fold in a genome [43],[44]. Krishnadev et al. [49] performed a similar study for the similarity graph of protein chains instead of protein domains. They also found scale-free behavior at large enough similarity threshold. They used spectral analysis of the adjacency matrix to partition the graph into clusters.

In contrast to these previous approaches, the graph presented here is not a preliminary step for clustering, but it represents the significant similarity relationships for which clustering is not justified. These relationships not only allow to recover relationships present in expert classifications, such as splitted superfamilies and folds, but also allow to treat on the same ground the cross-fold relationships discussed by several authors, which go beyond expert classifications.

**Figure 9:**
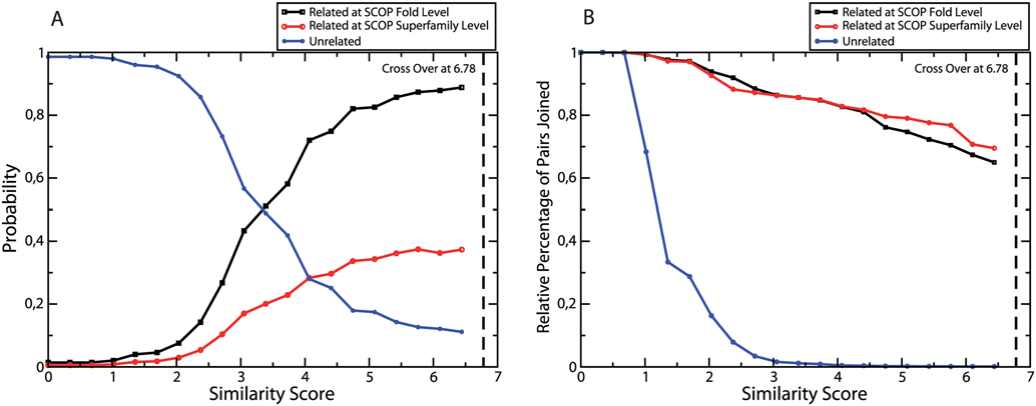
For networks of clusters in the automatic classification joined with the similarity threshold represented in the horizontal axis, we plot in (A) the fraction of links joining clusters that contain two proteins from the same SCOP superfamily (a), the same SCOP fold (b), or different folds (c), respectively; in (B) we plot the probability that a link exists for a pair of clusters of type (a), (b), and (c). In (A), we see that, for 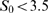, the majority of links are from clusters unrelated in SCOP.

We construct the similarity network by connecting the clusters of the automatic classification that have significant structural similarity. As the similarity threshold is decreased, more and more clusters are connected. Pairs of clusters containing structures from a superfamily splitted in the automatic classification get unified in the network. We measured the probability that a pair of domains is joined in the network as a function of the similarity threshold, distinguishing pairs of domains from the same superfamily, from the same fold, or from different folds. (see Figure 9). Only for similarities as low as 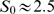, more than 90% of the domains in the same superfamily are joined. However, already for similarities 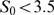 the majority of the joined domains are from different folds. A reasonable threshold for significant structure similarity, mostly corresponding to pairs of different folds, seems to be 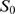 between 3 and 4. Results presented here are obtained using 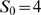 as threshold for significant structure similarity.

**Figure 10:**
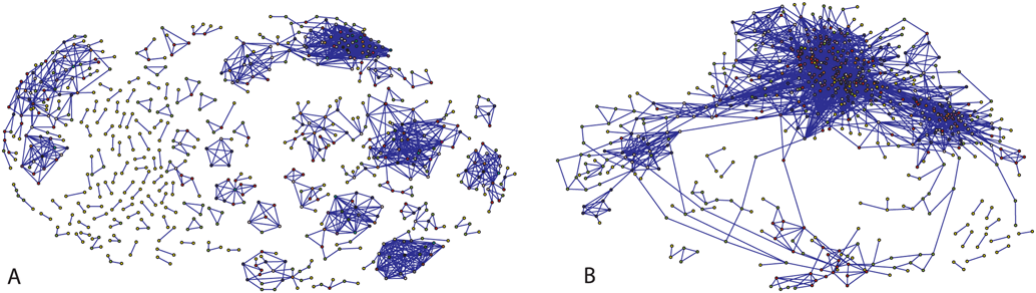
Networks of protein clusters similarities. (A) High similarity clusters (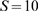) linked using as a threshold the cross-over similarity, 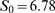. (B) Cross-over clusters (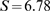) linked below the high transitivity regime, up to 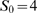.

A visual representation of such a network is shown in Figure 10B. One can see that almost all of the structure space is connected, but there is still some structure appearing. If we use a higher similarity threshold but still below the cross-over, such as 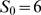, the resulting network contains several linear motifs clearly expressing transitivity violations, with 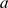 connected to 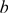, 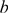 to 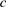, 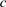 to 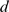, and so on, but without direct connection between 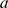 and 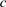 or 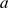 and 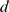. For comparison, we also show in Figure 10A the network constructed joining clusters at high similarity before the cross-over point (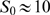) using as threshold the cross-over similarity, 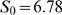. This network presents many regions with high density of links, representing clusters that have still to be joined,

In the context of network analysis, the transitive property studied in this paper is analogous to the clustering coefficient (see Methods). Clustering coefficient equal one means that the network is transitive, i.e., if 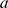 is connected with 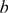 and 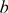 is connected with 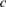, also 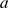 is connected with 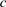. The high siilarity network obtained before the cross-over point has a high mean clustering coefficient equal to 0.69, which decreases to 0.36 for the network after the cross-over. In general, as one could expect, the clustering coefficient increases with the similarity threshold 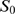 (see Figure S1). However this increase is smooth, so that we can not use the clustering coefficient to detect the cross-over point.

Interestingly, the network allows not only to recover similarity relationships at the superfamily and fold level that are below the threshold for clustering, but it may also help to discover new evolutionary or functional relationships that are not contained in SCOP or CATH. For instance, in a recent paper Xie and Bourne proposed a new method to detect remote evolutionary relationships based on the structure similarity of the active site [50]. Using this method, they confirm a previously proposed evolutionary relationship between SCOP superamily Phosphoenolpyruvate carboxykinase (PCK) and the P loop containing nucleotide triphosphate hydrolase (NTH) superfamily. The PCK domain 1ayl_1 used as a seed by Xie and Bourne is joined in the automatic classification with domains 1knxa2 and 1ko7a2, which are classified in SCOP in the PCK superfamily but are classified in CATH in the NTH superfamily. The automatic classification supports the CATH classification. This cluster has a single significant structural link, with average similarity 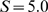, with a cluster containing only domains classified in the NTH superfamily in both CATH and SCOP, and through this link another step connects it to many other clusters in the NTH superfamily or in the NTH fold. The relevant part of the network is represented in Figure S4, from which it is clear that the structurally consistent clusters joined in a network give a richer evolutionary information than a unique fold.

In order to complement structure information with sequence information, we constructed the network connecting clusters that have members belonging to the same superfamily. The networks based on sequence and structure similarity can be accessed at the url http://ub.cbm.uam.es/research/ProtNet.php

#### Transitivity violations and protein modularity

**Figure 11:**
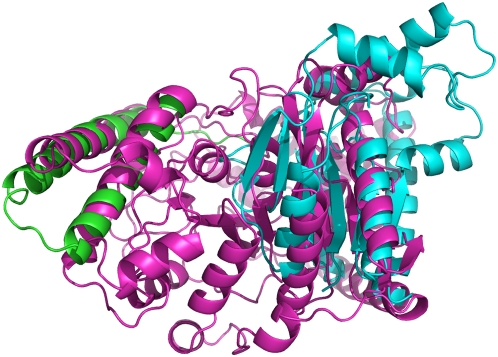
Example of three domains that violate transitivity with 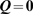. They are joined after the cross-over point in the network built using similarity threshold 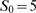. The ASTRAL codes are d1mt5a_ (a), d1bif_1 (b) and 1b3qa1 (c). The bigger domain d1mt5a_ (red) links in the network the two smaller domains, which deviate considerably from each other as they don't share any significant part of structure between them. It holds 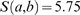 (red and blue), 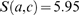 (red and green) and 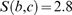 (blue and green), which violates transitivity.

To investigate protein modularity, we studied the triangles that violate transitivity for a specific threshold 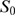, in the sense that 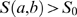, 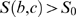, but 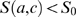. For such triangles, we tested whether the regions of the intermediate structure 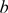 having a good match with structures 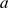 and 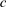 are the same or they are different, by measuring the overlap between these two regions as
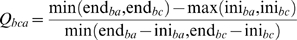
(4)where the initial and final residues of the matching regions are denoted as 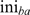, 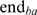, 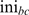 and 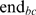, respectively. The value 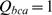 means that all three structures all share the same core over which they are similar. In contrast, the value 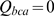 means that the intermediate structure 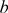 shares completely different fragments with structures 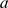 and 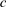. This is the most dangerous case for clustering algorithms, which can run the risk to join two structures that do not share any common region. One such example, with ASTRAL codes d1mt5a_, d1bif_1 and d1b3qa1, is shown in Figure 11.

**Figure 12:**
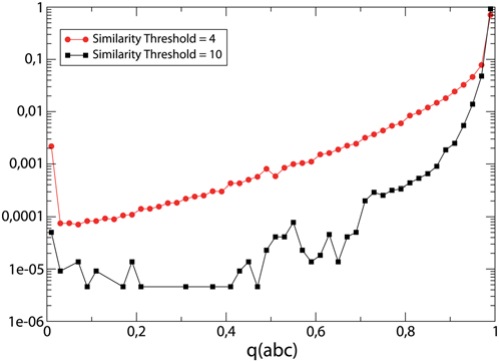
For networks defined through the condition 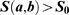, with 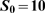 and 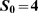, respectively, and for all triangles that violate the transitive property, i.e., 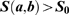, 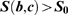 and 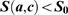, we measured the overlap 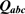 between the two relevant matches of the intermediate structure 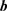, Eq. (4). The peaks of the distribution at 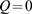 and 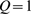 correspond to matches over completely different and exactly the same region of protein 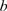, respectively.

The distribution of the fragment overlap 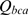 is bimodal, with peaks at 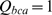 and 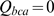 (see Figure 12). However, triangles with 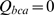 are very rare for large similarity 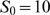, where they may correspond to errors in domain decompositions, whereas they become more frequent for similarities below the cross-over point.

Thus, beyond the cross-over point it is likely to find severe violations of transitivity in which two significant matches 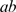 and 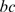 fall in two completely different regions of protein 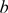, consistent with the idea that transitivity violations and the consequent continuity of protein structure space stem from the modularity of proteins. These significant and disjoint partial matches offer a way to operatively define substructures below the domain level. A more detailed study of substructures based on their recurrence will be presented elsewhere.

## Discussion

### Transitivity Violations

As for all problems for which hierarchical clustering algorithms are applied, for clustering protein structures it is of key importance to determine up to which point the clustering is justified. We propose to test the internal consistency of a clustering method based on a similarity measure by testing the transitive property, which requires that whenever 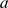 is similar to 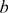 and 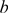 is similar to 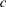, then 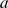 must be similar to 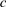. Only if the transitive property holds a hierarchical classification can be unambiguously built. If the transitive property is violated for an extensive number of triangles, hierarchical clustering is frustrated [38], and we expect that there is a very large number of unrelated and almost optimal classifications, in each of which a similar number of similarity relationships are violated. We proposed here Eq. (1) to quantify the violations of transitivity of a group of three elements, and Eq. (2) to quantify the violation of transitivity when two clusters are joined.

Transitivity violations as defined here occur either when a pair of domains is joined below the similarity threshold, or when a pair is separated above the same threshold. Another definition, common in the context of sequence comparisons, considers that transitivity is violated only when pairs are separated above threshold. This definition is motivated by the fact that significant sequence similarity demonstrates almost certainly an evolutionary relationship, whereas the lack of similarity does not exclude it. With this definition, the single linkage algorithm does not produce any transitivity violation, since it joins all pairs above threshold. In fact, the term transitivity is often used as a synonymous of single linkage clustering.

Nevertheless, several reasons make the definition of transitivity adopted here more suitable in the context of structure classification. The first reason also applies to sequence comparisons, and it is based on protein modularity. If a domain 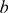 is made of two fragments 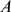 and 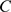, with 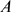 similar to domain 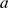 and 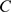 similar to domain 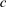, single linkage will infer a non existing relationship between 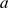 and 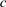. Indeed, for applying single linkage clustering to the triangle 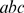, one has to check whether the fragment overlap 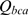, Eq. (4), is also significant. Secondly, single linkage joins many structures that are not significantly similar, producing clusters that are not structurally consistent. These clusters may lack a common core, as it is often found applying multiple structure alignment algorithms to SCOP and even more CATH superfamilies. For the goal of modelling, it may not be convenient to join structurally dissimilar domains in the same fold, since this would increase the likelihood of selecting wrong templates. The study of structure evolution is made more difficult when structural variation is hidden inside a very diverse cluster, whereas well defined clusters connected by links expressing evolutionary relationships may represent a better framework for the study of structure divergence.

### Cross-Over from Discrete Sets to Continuous Space

We have observed that the transitivity violations grow while the clustering algorithm joins protein domains into clusters. Interestingly, in all instances that we studied we have found a cross-over between two regimes of slow and fast increase of transitivity violations.

1. At high similarity, transitivity violations grow slowly as the clustering algorithm proceeds, and domain size does not vary very much within a cluster. Clusters in this regime mostly correspond to subsets of SCOP superfamilies. Therefore, most domains in the same cluster are related through gene duplication and subsequent divergence, which justifies to classify related domains on a tree.
2. At low similarity, transitivity violations grow rapidly as the clustering algorithm proceeds, and domains in the same cluster differ substantially in size. Many pairs in the same cluster are related through partial substructures.

We propose that the cross-over in transitivity violations is an intrinsic point to stop the automatic classification. Lower similarity relationships should be represented as a network rather than a tree.

### Influence of the Methodology

The method that we presented requires several arbitrary choices. In order to test its robustness, and the influence of the parameters, we have studied at least two alternatives for each of these choices. Qualitatively similar results were obtained for several similarity scores computed on two different alignments obtained with a local and a global version of the MAMMOTH algorithm. Both alignment algorithms were developed at our group. We did not test whether alignments obtained with algorithms developed by other groups, such as DALI, yield different conclusions, as they might do.

In all cases that we tested, we have observed a cross-over in transitivity violations, finding that most of the clusters at the cross-over point correspond to subsets of SCOP or CATH superfamilies. However, the exact location of the cross-over point and the quality of the clustering, as assessed through the clustering coefficient and through the mean value of the transitivity violations, varies for different choices.

Although we do not aim at reproducing SCOP or CATH, which we believe is impossible, we recognize that these expert classifications have important merits. It is therefore noteworthy that the highest clustering coefficients and lowest transitivity violations tend to be associated with scores that are better compatible with SCOP or CATH classifications.

The first important choice is the structure alignment algorithm. Computationally, structure alignment is an NP-complete problem, and even if it were exactly solved different algorithms would differ, since they optimize different scores. We used two versions of the algorithm MAMMOTH that are quite different, since one optimizes local superimmposition of heptamers whereas the second one, MAMMOTH-mult, otpimizes the global structure superimposition, achieving alignments with better PSI and contact overlap. Despite this important difference, the results obtained with the two methods are rather similar.

The similarity measure used is probably the most relevant choice, and we tried several of them. We obtained better results with the contact overlap than with measures that score the optimal spatial superimposition of the two structures, which are used in the standard MAMMOTH score. We conjecture that the contact overlap is a better measure than the PSI for clustering protein structures because of three reasons: (1) It does not assume that there is an optimal rigid body superimposition between the two structures. In doing so, it implicitly allows for flexible superimpositions, which might be better suited for detecting evolutionary relationships [51]–[54]. (2) It weights the residues in the core of the protein more than loop residues, since the former have a larger number of contacts. (3) The parameter it depends on, i.e., the threshold at which two residues are considered in contact, has a physical meaning in terms of interatomic interactions, and it is therefore less arbitrary than the tolerance parameter of the PSI, i.e., the threshold below which two residues are considered to be superimposed.

Similarity scores based on structure superimposition typically need a tolerance threshold to decide whether two residues superimpose. We tested the TM score [42], which uses a length dependent threshold that makes this score almost independent of the size of the aligned proteins. The results obtained with this score are very similar to those obtained with the contact overlap. In contrast, the percentage of structure identity (PSI) adopts a fixed tolerance threshold, usually chosen as 4Å . To study the effect of this parameter, we repeated our numerical experiments with a more tolerant threshold of 6Å . Not surprisingly, the more tolerant similarity measure makes the space more continuous, decreasing the clustering coefficient and increasing the transitivity violations. Therefore, the cross-over from the discrete to the continuous regime occurs at higher similarity, which means that protein domains are splitted into a larger number of clusters. In this case as well, the cross-over is clear and the clusters at the cross-over are mainly subsets of superfamilies.

All measures, except the TM score, must be normalized in order to make them independent of the length of the aligned proteins. We implemented this through a length dependent Z score, as in the original MAMMOTH score. The drawback of the Z score is that not only it makes the similarity of unrelated proteins almost independent of length, but at the same time it reduces the similarity of related proteins with short length. In this way, the similarity of related proteins depend on their length and not on their evolutionary divergence, which makes the Z score an unsuitable measure for evolutionary analysis. This drawback does not occurr with the TM score, although this does not necessarily imply that it is a suitable measure for evolutionary analysis.

Last, we have to decide which clustering algorithm we use. If we adopt the definition of transitivity proposed in the present work, the average linkage algorithm has to be preferred over both single linkage and complete linkage. In fact, average linkage reduces the combination of splitting and overunification errors, whereas single linkage only eliminates splitting errors, since it joins all pairs above the similarity threshold, and the complete linkage eliminates overunification errors, since it separates all structures that are below the similarity threshold. Interestingly, from our analysis it turns out that the main difference between SCOP and CATH is that the latter uses single linkage, while the former uses some procedure effectively similar to average linkage.

As a last remark, we note that there is some analogy between our method, which uses transitivity violations to detect the point at which hierarchical clustering is not justified, and the bootstrap method that scores the significance of each cluster in a tree. Nevertheless, there are also important differences. Besides the fact that bootstrap is computationally much more cumbersome than our method, for obtaining a classification with the bootstrap method we would have to fix a threshold bootstrap probability to accept one cluster, whereas the cross-over that we obtain with our method arises in a natural way without fixing an arbitrary threshold.

### Perspectives for the Automatic Classification of Proteins

The existence of two regimes of transitivity violations, and the fact that the automatic classification at the cross-over point mostly consists of sets of SCOP or CATH superfamilies are the main results of this work. They are robust with respect to changes in the clustering algorithm, the similarity measure, the set of protein domains that we automatically classify, and the accuracy of the alignment algorithm. These results suggest that it is possible to automatically and objectively define an equivalence class for protein domains up to the similarity corresponding to the cross-over point.

Clusters in the automatic classification are structurally more consistent than SCOP folds or CATH topologies, mainly because of two reasons. (1) In the automatic classification, almost 15 percent of superfamilies are split into structurally divergent clusters, indicating that there can be important structural changes in protein evolution [32],[33],[36]. Interestingly, domains in split superfamilies tend to have larger size difference between each other, suggesting that insertions and deletions play an important role for structural divergence, consistent with recent analysis [55],[56]. (2) Only 44 percent of the pairs of domains in different SCOP superfamilies and the same SCOP fold are joined in the automatic classification. This percentage becomes much smaller for CATH (less than 11 percent), whereas 68 and 66 percent of the pairs in the same SCOP or CATH superfamily are joined in the automatic classification The similarity between most of the pairs that are not joined is significant, but it is at the level where transitivity violations are large and a network fits the data better than a classification. Our analysis thus suggests that CATH and SCOP classify proteins up to similarities that are below the cross-over of transitivity violations. The same is possibly true for the automatic FSSP classification as well, where proteins are classified in the same fold if the Z score of their similarity is above 2. This is the smallest threshold at which the structures compared are significantly related. Here we also use a Z score, but we find that the cross-over point is at 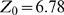 implying that the transitive property is severely violated at the similarity level 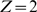.

An indication that the fold defined in expert classification may not correspond to an intrinsic similarity level is that CATH and SCOP neatly agree at the level of superfamily, as assessed through the weighted kappa measure, but they disagree between each other at the level of fold even more than they disagree with the automatic classification, when the proper clustering algorithm is used. Indeed, the main difference between SCOP and CATH at fold level is that SCOP uses a procedure effectively similar to the average linkage algorithm, whereas CATH uses the single linkage algorithm, which does not penalize the joining of structurally distinct domains, resulting in clusters that are structurally very diverse.

Furthermore, we have shown that the structural diversity within a SCOP fold is larger if the fold was defined since longer time, suggesting that the criteria underlying the definition of fold may change through time. Classifications are very useful, but the present analysis supports the view that the low similarities at the fold level are better represented as a network rather than as a tree.

### Possible Improvements of the Automatic Classification

The comparison between the automatic and the expert classifications also indicates that the automatic classification can be improved along three lines.

First, in the present study we considered protein domains as defined in the SCOP and CATH classifications. However, proteins are split into domains in the two schemes in a rather different way. In particular, some domains defined in the SCOP classification appear by visual inspection to consist of more than one domain. An incomplete domain partition can be an important source of transitivity violations and consequent errors in an automatic classification of protein structures. We are developing a new automatic method for decomposing proteins into domains based on their recurrence in a database of unrelated structures, similar to the method proposed by Holm and Sanders [57]. The domains obtained in this way will be subject to further decomposition based on their structure, to obtain a set of domains to which we will apply our clustering procedure.

Secondly, our method tends to split superfamilies constituted of short domains. Some of these splitting appear to be due to the dependency of the similarity score on the protein length. The raw similarity score, either PSI or contact overlap, is transformed into a Z score in order to reduce as much as possible the dependency of the score of unrelated structures on their size. Our results show that the classification deteriorates if this normalization is not properly performed. However, due to this normalization the similarity score corresponding to identical structures decreases for decreasing domain size, which makes it more difficult to cluster together short proteins. In order to overcome this problem, it would be very helpful to define a similarity score that is independent of domain size both for unrelated and for closely related structures. This will be presented in a forthcoming work.

Third, we found 63 over 779 clusters that contain protein domains defined by SCOP curators as different folds (although 27 of these clusters are homogeneous in terms of CATH topologies). The distribution of structure similarity suggests that several of the foreign domains appearing in clusters that are mostly from another fold are characterized by low mean similarity, and that it could be possible to “clean” the clusters of the automatic classification. Preliminary results indicates that this strategy is promising.

### Protein Domain Networks

Significant sequence or structure similarity below the threshold for clustering [14],[15] constitutes a very valuable information for evolutionary or functional studies. In the CASP and SCOP database, these significant cross-fold similarities are not available. We present this information in the form of two networks with structure-based and sequence-based links between the clusters of the automatic classification. In this way, we can recover not only superfamily and fold relationships that are not present in the automatic classification, but also new relationships that are not reported in expert classifications.

### Two Modes of Protein Evolution?

As a concluding remark, we note that the two regimes of transitivity violations that we found can be related with two modes of protein domain evolution. In the regime of large structure similarity, transitivity violations are small, related domains are similar in size, and 95 percent of them contain domains from a single CATH or SCOP fold, whereas 86 percent contain evolutionarily related domains from the same superfamily. These results indicate that most of the domains with structure similarity above the cross-over are evolutionarily related through gene duplication and divergent evolution. Moreover, domains in different superfamilies but same fold can not be excluded to be evolutionarily related, and some careful studies have been able to demonstrate this common origin also in the absence of a clear signal from sequence similarity, as in the case of the study of TIM-barrels conducted by Nagano et al. [58]. This view also agrees with the results by Deeds et al. [59], who tested models of convergent and divergent evolution using statistical properties of protein structural clusters, finding that the data support divergent evolution [60]. We summarize these findings saying that, for large similarity, protein domain evolution is mostly uniparental.

On the other hand, similarities below the cross-over of transitivity violations are often due to partial substructures, and the typical size difference between related domains raises from 20 to 40 residues, indicating the occurrence of large insertions and deletions when the related domains belong to the same superfamily. These are clues of multi-parental evolution, proceeding through the assembly of new polypeptide fragments. This hypothetical mechanism has been proposed by Lupas et al. for the evolution of early protein domains through assembly of small peptide fragments [28]. Our findings suggest that it can also be extended to more recent evolution, consistent with another recent study [15]. In this regime the domain structure space should be regarded as continuous, and significant structure similarity should be described as a network rather than a tree.

These considerations parallel recent considerations about the classification of organisms on the tree of life [61]. Speciation and evolutionary divergence generate a tree of species, which can be reconstructed by estimating the time of divergence from the molecular sequences of their genes. In order to do this, one has to use a proper sequence distance, approximately ultrametric, which makes species classification possible on a rigorous basis. Nevertheless, this view of the tree of life has been recently challenged by the discovery of the high rate of horizontal gene transfer in genome evolution. Due to horizontal gene transfer, genome evolution is multiparental, and genes that have been subject to gene transfer can not be used to reconstruct the phylogenetic tree. The extensive presence of horizontal gene transfer in evolution has led Doolittle to propose that the evolutionary relationships between organisms should be regarded as a net of life rather than a tree [61]. The present work suggests that, in the context of protein domain evolution, a tree scenario of uniparental divergent evolution is suitable to represent high similarity relationships, but a pluriparental network emerges for more remote relationships.

## Methods

### Datasets

We have used two non redundant sets of protein domains. The first set was obtained from the ASTRAL 40 database, in which no pair has sequence similarity larger than 40%. We used the SCOP version 1.65 and selected only domains from the four main SCOP classes, all 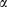, all 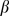, 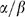 and 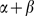. The second set is the non redundant set of domains from the CATH classification, with sequence similarity smaller than 35%. Also in this case we excluded domains outside the four main classes. The final number of domains was 5041 for the SCOP set and 7073 for the CATH set.

### Consensus Set between CATH and SCOP

In order to select a set of domains consistently defined in SCOP and CATH, we aligned with BLAST [62] the sequences of domains in the non redundant ASTRAL40 database against domains in the non redundant CATH database at 35% sequence identity. We identified two domains to be equivalent if their BLAST evalue was smaller than 10^−3^, with sequence identity larger than 75%, and their size differed by less than 10%. In this way we have obtained a set of 2890 non redundant domains classified in 779 SCOP superfamilies, 466 SCOP folds, 885 CATH superfamilies and 473 CATH topologies.

### Similarity Scores

We performed pairwise structure alignments using either the program MAMMOTH [41], which is the fastest program of protein structure alignment that we know, or its multiple alignment version MAMMOTHmult [39], which is a bit slower but much more accurate.

The MAMMOTH similarity score is based on the number of aligned residues that are closer than 4Å after optimal spatial superimposition of structures 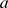 and 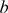, 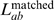. This is transformed into a percentage of structure identity (PSI) dividing it by the length of the shortest structure,
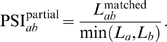
(5)



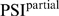
 equals one if the two structures coincide over the length of the shorter one. There is no penalization for additional residues in the longer structure, i.e., the score is sensitive to good partial matches and we call it partial PSI. However, the fact that the score does not penalize inserted regions may lead to join domains with very large length difference. To tackle this problem, we also defined the total similarity score, which penalizes regions in the larger structure that are not matched by the short one:
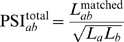
(6)

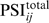
 equals one only if the match completely covers the longer protein.

Third, we adopted the contact overlap, which counts the fraction of contacts in common between two aligned structures 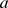 and 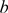. Also this score is normalized in such a way to penalize partial matches. We defined the contact matrix 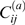 of protein 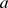 such that 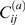 equals one if two heavy atoms of residues 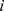 and 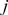 are closer than 4.5Å and 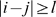, and zero otherwise. We considered two cases, 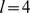 and 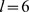. In this last case, intrahelical contacts are not considered. Denoting by 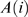 the residue in structure 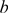 aligned with residue 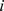 in structure 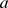, the contact overlap can be written as
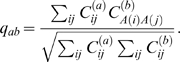
(7)


The main qualitative difference between the contact overlap and the PSI is that in the contact overlap superimposed residues in the core of the protein, which form many contacts, receive a larger weight.

It is crucial for protein structure classification that the distribution of the similarity score used is almost independent of the length for comparisons of unrelated proteins. The MAMMOTH score takes care of this by normalizing the PSI in such a way that the distribution of the normalized PSI is almost independent of size for unrelated pairs:
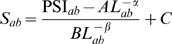
(8)where 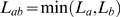 in the case of the partial PSI, and 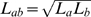 in the case of the total PSI. In the case of the overlap, we also used 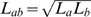 as a normalization. The exponents 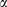 and 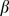 depend on the raw similarity score and on the alignment algorithm used, and they were determined by fitting the mean and standard deviation of the PSI of unrelated structures having 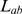 in some given interval, using the best fit between a Gaussian fit or an Extreme Value statistics fit (see Table 4).

**Table 4:** Size normalization of similarity scores.

Score	Normalization	Alignment	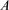	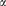	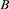	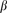
PSI partial	EV	Pair	5.97	0.720	0.920	0.634
PSI partial	EV	Mult	5.73	0.714	0.860	0.622
PSI total	EV	Pair	6.48	0.722	0.972	0.662
PSI total	EV	Mult	5.62	0.729	0.961	0.659
Overlap	Gauss	Pair	0.375	0.535	1.340	0.676
Overlap	Gauss	Mult	0.752	0.576	1.874	0.773

The reported parameters were used to normalize the raw scores according to Eq. (8).

Using Gaussian statistics, we fit
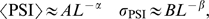
(9)and using Extreme Value statistics, we fit
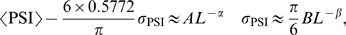
(10)


The domain similarity score of domain 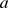 in cluster A is defined as the average pairwise similarity between domain 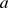 and all other domains in the cluster,
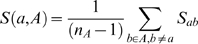
(11)


### Clustering Algorithms

We programmed and tested three hierarchical clustering algorithms: average linkage [63], single linkage and complete linkage. Starting from each element being a separate cluster, at each step 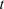 all algorithms join the two most similar clusters 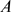 and 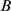, and compute the similarity between the new combined cluster and all other clusters in a way that depends on the clustering algorithm.

With **average linkage**, the combined similarity is computed as the average similarity with the two joined clusters,
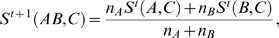
(12)where 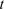 labels the step of the algorithm, A and B are the clusters that are joined, 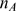 and 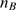 is the number of elements they contain, AB denotes the new composite cluster, and C is any other cluster. Note that this updating rule is equivalent to computing the new similarity score as the average between the similarity between all pairs of elements from the cluster C and the cluster AB.

With **single linkage**, the combined similarity is the largest similarity in the set, so that two sets are joined if at least one pair of elements is above threshold
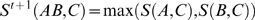
(13)


With **complete linkage**, the combined similarity is the smallest similarity in the set, so that two sets are joined if all pairs of elements are above threshold
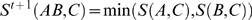
(14)


### Ultrametricity

An ultrametric set is a set 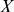 with an associated distance measure 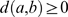 where every triplet of points 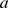, 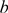 and 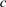 fulfils a property stronger than the ordinary triangular inequality: each side of a triangle is smaller than the larger between the other two sides, i.e., 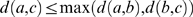. This implies that the two longer sides must be equal. In particular, for an ultrametric set and for every threshold 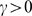, it holds that if 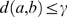 and 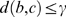, then 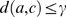. Consider now the cluster containing all elements within a distance 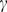 from element 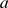, 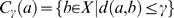. It is easy to see that, for every pair of points 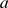 and 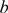, either 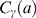 and 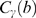 coincide, or they do not share any point. Therefore, 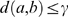 is an equivalence relationship, since if 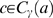 then it must also be 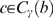, and the set of points can be considered discrete.

### Clustering Coefficient

A concept related to transitivity in the context of networks is the clustering coefficient, which can be computed through the formula
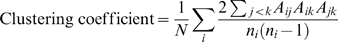
(15)where 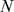 is the number of nodes in the network, labelled as 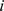, 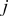 and 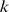, 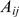 is the adjacency matrix (one if 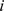 and 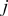 are joined, zero otherwise), 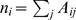 is the number of neighbors of node 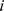, and the clustering coefficient of node 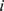 is the fraction of pairs of its neighbors 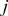 and 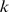 that are neighbors between each other. If the clustering coefficient is one for all nodes, connections on the network define an equivalence relationship.

We have computed the clustering coefficient for the network obtained by joining domains with similarity 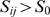, for various values of 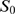. To compare different similarity measures, we have plotted the clustering coefficient versus the number of clusters obtained through single linkage clustering with the same threshold 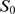.

### Detecting the Cross-Over Point

For detecting the cross-over point of transitivity violations (TV), we first measure TV at each step of the clustering algorithm using Eq. (2). We then perform two-pieces exponential fits of TV versus the step 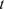, as 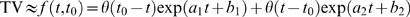, where 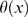 is zero for negative 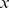 and one otherwise. Fits are performed for all possible cross-over points 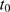, and their quadratic error is measured as
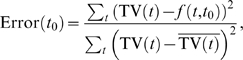
(16)where 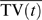 is the mean value of TV. To find the optimum 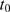 in a robust way, we perform a cubic fit of the error function in an interval 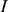 centered around the step 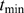 yielding the minimum error, and such that 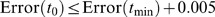 for all 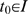. The analytic minimum of this cubic fitting is then selected as the best first estimate of the cross-over point.

The last points in the 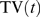 curve, where the transitivity violations approach the maximum possible value, are very badly fitted through the two-pieces fit. Therefore, we refined the estimate of the cross-over point by removing the outliers of the optimal fit, with the conditions that a point is removed if its residual with respect to the optimal fit is more than three times larger than the median, which is the condition used to define type-1 outliers. We then apply the procedure described above to the reduced set of points, and we determine the cross-over point at which the clustering is stopped.

### Weighted Kappa

We assessed the agreement of two classifications through the weighted kappa measure [64], which uses as reference the expected agreement for two independent classifications with the same number of relationships. We define 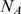 (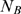) the number of related pairs in classification 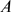 (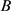) of the same 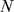 objects, with 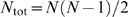 pairs in total. If 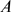 and 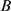 are independent, the number of pairs that are either related or unrelated in both 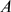 and 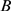 is given by
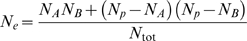
(17)


We compare this number to the observed number of pairs that agree,
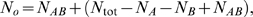
(18)where 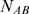 is the number of pairs that are related in both classifications. >From this number, the weighted kappa is computed as
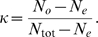
(19)


A value of zero means that two classifications are as related as independent classifications, one means that the two classifications coincide. Using the weighted kappa, we have compared the classification obtained at every step of the clustering algorithm with the manual classifications of CATH and SCOP at the superfamily and the fold level.

Notice that the weighted kappa can be decomposed into the contributions of related and unrelated pairs as follows:
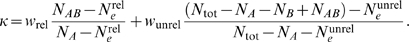
(20)where 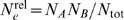 is the number of pairs related in both classifications expected by random, 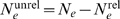, and the weights are 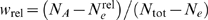 and 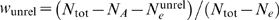 for related and unrelated pairs, respectively.

### Network Analysis

For the sake of illustration, we have represented two domain similarity networks obtained before and beyond the stopping point of the automatic classification.

Two networks were constructed by considering each cluster as a node, and connecting nodes with 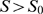. In the first case, we used clusters obtained before the cross-over point of the average linkage algorithm using a high similarity threshold 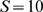, and we connected them if 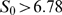, which is the similarity at the cross-over point. In the second case we used clusters generated at the cross-over point and we connected them with 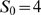. The networks have been visualized using the Pajek software [65].

### Other Methods

To visualize spatial superimpositions, we used the multiple structure allignments program MAMMOTHmult [39] in combination with the Pymol software.

## Supporting Information

Figure S1 Clustering coefficient for three different similarity measures. The clustering coefficient is computed for networks in which domains with similarity above S_0_ are connected, and it is plotted as a function of the number of clusters obtained with single linkage clustering of the same network.
(0.02 MB PDF)

Figure S2 Transitivity violations versus the step of the clustering algorithm for three different clustering algorithms. The smallest violations are obtained with the average linkage algorithm.
(0.19 MB PDF)

Figure S3 Agreement between the classifications obtained with different clustering algorithms at the same step. The best agreement is between single linkage and complete linkage.
(0.05 MB PDF)

Figure S4 Network of protein clusters joining superfamilies NTH and PCK. Xie and Bourne confirmed a previously proposed evolutionary relationship between a member of SCOP superamily Phosphoenolpyruvate carboxykinase (PCK), with code 1ayl_1, and the P loop containing nucleotide triphosphate hydrolase (NTH) superfamily. PCK domain 1ayl_1 is joined in the automatic classification with domains 1knxa2 and 1ko7a2, which are classified in SCOP in the PCK superfamily but are classified in CATH in the NTH superfamily. The automatic classification supports the CATH classification. This cluster has a single significant structural link, with average similarity *S* = 5.0, with an cluster containing only domains classified in the NTH superfamily in both CATH and SCOP, and through this cluster another step connects it to many other clusters in the NTH superfamily or in the NTH fold. Here we represent the relevant part of the network. The hybrid cluster containing domain 1ayl_1 is close to the upper left corner. Links denote significant structure similarity between clusters (*S*>4.0), and they are coloured red if the two joined clusters contain domains in the same superfamily according to both SCOP and CATH, green if they are in the same superfamily only according to CATH, blue if they are in the same fold according to either SCOP or CATH, and black if there is no pair in the same fold. The figure supports the view that the structurally consistent clusters joined in a network give a richer evolutionary information than a unique and structrally diverse fold.
(0.02 MB PDF)
