# Supplementary material for: Cross-Over between Discrete and Continuous Protein Structure Space: Insights into Automatic Classification and Networks of Protein Structures
Source: PLoS Comput Biol. 2009 Mar 27;5(3):e1000331. doi: 10.1371/journal.pcbi.1000331 (PMC2654728; doi:10.1371/journal.pcbi.1000331)
Supplement: Figure S1 — Clustering coefficient for three different similarity measures. The clustering coefficient is computed for networks in which domains with similarity above S0 are connected, and it is plotted as a function of the number of clusters obtained with single linkage clustering of the same network. (0.02 MB PDF) [file pcbi.1000331.s001.pdf]

# Supporting Figure 1

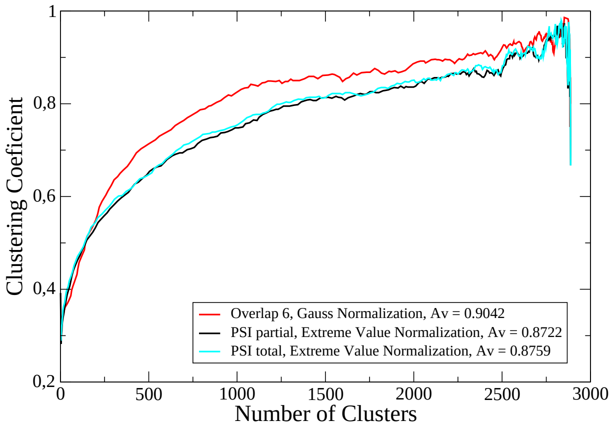


Clustering coefficient for three different similarity measures. We build networks by joining domains with $S_{ij} > S_0$ and plot their clustering coefficient versus the number of clusters obtained with single linkage clustering of the network. The smaller $S_0$, the fewer clusters there are and the smaller is the clustering coefficient.
