# Supplementary material for: Cross-Over between Discrete and Continuous Protein Structure Space: Insights into Automatic Classification and Networks of Protein Structures
Source: PLoS Comput Biol. 2009 Mar 27;5(3):e1000331. doi: 10.1371/journal.pcbi.1000331 (PMC2654728; doi:10.1371/journal.pcbi.1000331)
Supplement: Figure S2 — Transitivity violations versus the step of the clustering algorithm for three different clustering algorithms. The smallest violations are obtained with the average linkage algorithm. (0.19 MB PDF) [file pcbi.1000331.s002.pdf]

Supporting figure 2

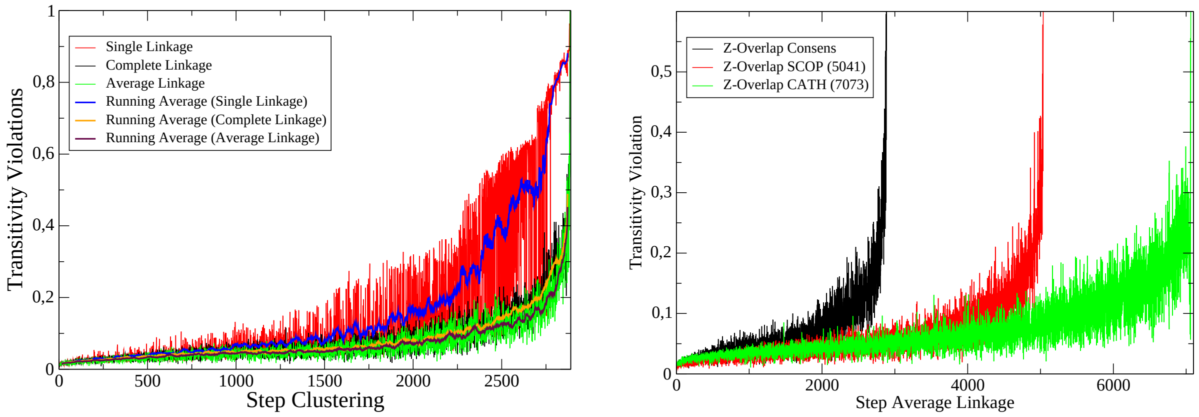

Left plot: Transitivity violations for the single linkage, average linkage and complete linkage algorithms. Right plot: Transitivity violations for three sets of domains with 2890, 5041 and 7073 domains respectively.
