# Supplementary material for: Cross-Over between Discrete and Continuous Protein Structure Space: Insights into Automatic Classification and Networks of Protein Structures
Source: PLoS Comput Biol. 2009 Mar 27;5(3):e1000331. doi: 10.1371/journal.pcbi.1000331 (PMC2654728; doi:10.1371/journal.pcbi.1000331)
Supplement: Figure S3 — Agreement between the classifications obtained with different clustering algorithms at the same step. The best agreement is between single linkage and complete linkage. (0.05 MB PDF) [file pcbi.1000331.s003.pdf]

Supporting Figure 3

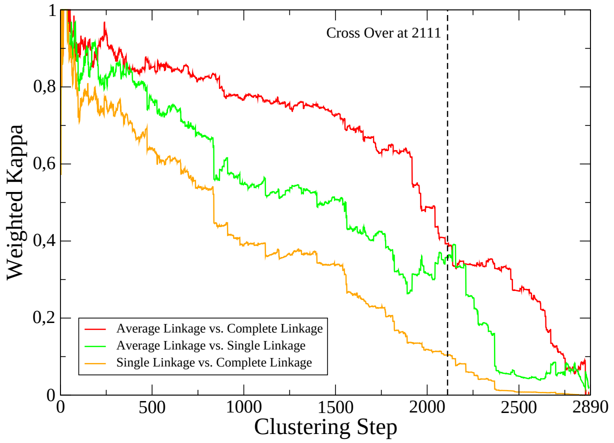

Agreement between the classifications obtained with different clustering algorithms at the same step. The best agreement is between single linkage and complete linkage. At the cross-over point, depicted as a vertical line, average linkage and single linkage have a local maximum in their weighted kappa.
