# Supplementary material for: Cross-Over between Discrete and Continuous Protein Structure Space: Insights into Automatic Classification and Networks of Protein Structures
Source: PLoS Comput Biol. 2009 Mar 27;5(3):e1000331. doi: 10.1371/journal.pcbi.1000331 (PMC2654728; doi:10.1371/journal.pcbi.1000331)
Supplement: Figure S4 — Network of protein clusters joining superfamilies NTH and PCK. Xie and Bourne confirmed a previously proposed evolutionary relationship between a member of SCOP superamily Phosphoenolpyruvate carboxykinase (PCK), with code 1ayl_1, and the P loop containing nucleotide triphosphate hydrolase (NTH) superfamily. PCK domain 1ayl_1 is joined in the automatic classification with domains 1knxa2 and 1ko7a2, which are classified in SCOP in the PCK superfamily but are classified in CATH in the NTH superfamily. The automatic classification supports the CATH classification. This cluster has a single significant structural link, with average similarity S = 5.0, with an cluster containing only domains classified in the NTH superfamily in both CATH and SCOP, and through this cluster another step connects it to many other clusters in the NTH superfamily or in the NTH fold. Here we represent the relevant part of the network. The hybrid cluster containing domain 1ayl_1 is close to the upper left corner. Links denote significant structure similarity between clusters (S>4.0), and they are coloured red if the two joined clusters contain domains in the same superfamily according to both SCOP and CATH, green if they are in the same superfamily only according to CATH, blue if they are in the same fold according to either SCOP or CATH, and black if there is no pair in the same fold. The figure supports the view that the structurally consistent clusters joined in a network give a richer evolutionary information than a unique and structrally diverse fold. (0.02 MB PDF) [file pcbi.1000331.s004.pdf]

# Supporting Figure 4

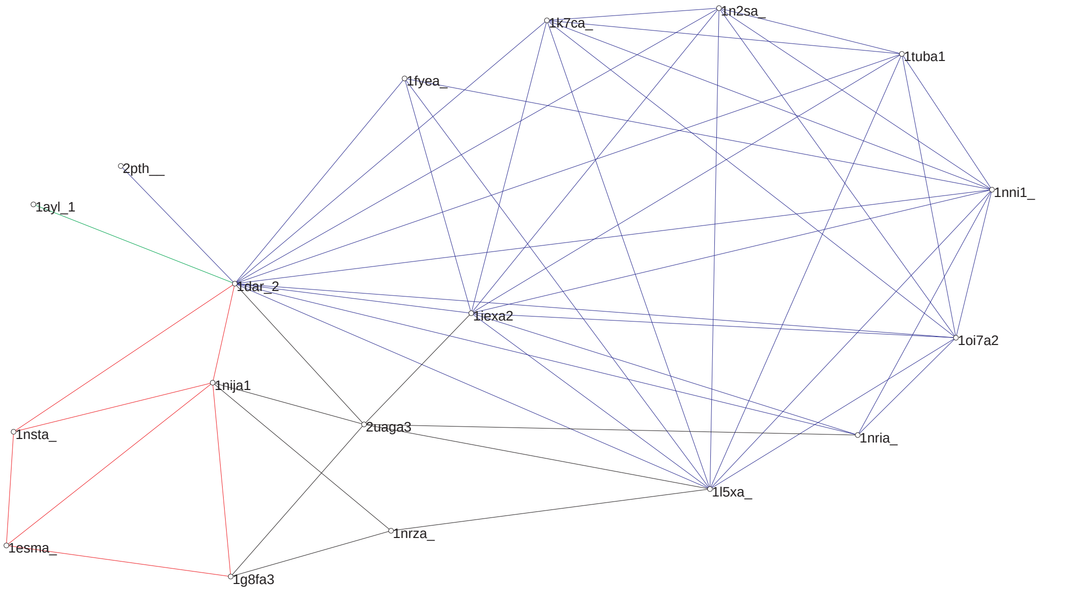


Network of protein domain clusters that contain the superfamilies NTH and PCK. Xie and Bourne confirmed a previously proposed evolutionary relationship between a member of the SCOP superamily Phosphoenolpyruvate carboxykinase (PCK), with code 1ayl_1, and the P loop containing nucleotide triphosphate hydrolase (NTH) superfamily. The PCK domain 1ayl_1 is joined in the automatic classification with the domains 1knxa2 and 1ko7a2, which are classified in SCOP in the PCK superfamily but are classified in CATH in the NTH superfamily. This cluster has a single significant structural link (average similarity $S = 5.0$) with a cluster containing only domains classified in the NTH superfamily in both CATH and SCOP, and through this cluster another step connects it to many other clusters in the NTH superfamily or in the NTH fold. Here we represent the relevant part of the network. The hybrid cluster containing the domain 1ayl_1 is close to the upper left corner. Links denote significant structure similarity between clusters ($S > 4.0$), and they are coloured red if the two joined clusters contain domains in the same superfamily according to both SCOP and CATH, green if they are in the same superfamily only according to CATH, blue if they are in the same fold according to either SCOP or CATH, and black if there is no pair in the same fold. The figure supports the view that the network of structurally consistent clusters gives a richer evolutionary information than the hierarchic classification into folds.
